# The value of arterial spin labelling in adults glioma grading: systematic review and meta-analysis

**DOI:** 10.18632/oncotarget.26674

**Published:** 2019-02-22

**Authors:** Amirah Alsaedi, Fabio Doniselli, Hans Rolf Jäger, Jasmina Panovska-Griffiths, Antonio Rojas-Garcia, Xavier Golay, Sotirios Bisdas

**Affiliations:** ^1^ Department of Radiology Technology, Taibah University, Medina, KSA; ^2^ Department of Brain Repair & Rehabilitation, Queen Square Institute of Neurology, University College London, London, UK; ^3^ Postgraduate School in Radiodiagnostics, Università degli Studi di Milano, Milan, Italy; ^4^ PhD Course in Clinical Research, Università degli Studi di Milano, Milan, Italy; ^5^ Lysholm Department of Neuroradiology, The National Hospital for Neurology and Neurosurgery, University College Hospitals NHS Trust, London, UK; ^6^ Department of Applied Health Research, University College London, London, UK

**Keywords:** glioma, arterial spin labeling, grading

## Abstract

This study aimed to evaluate the diagnostic performance of arterial spin labelling (ASL) in grading of adult gliomas. Eighteen studies matched the inclusion criteria and were included after systematic searches through EMBASE and MEDLINE databases. The quality of the included studies was assessed utilizing Quality Assessment of Diagnostic Accuracy Studies-2 (QUADAS-2). The quantitative values were extracted and a meta-analysis was subsequently based on a random-effect model with forest plot and joint sensitivity and specificity modelling. Hierarchical summary receiver operating characteristic (HROC) curve analysis was also conducted. The absolute tumour blood flow (TBF) values can differentiate high-grade gliomas (HGGs) from low-grade gliomas (LGGs) and grade II from grade IV tumours. However, it lacked the capacity to differentiate grade II from grade III tumours and grade III from grade IV tumours. In contrast, the relative TBF (rTBF) is effective in differentiating HGG from LGG and in glioma grading. The maximum rTBF (rTBFmax) demonstrated the best results in glioma grading. These results were also reflected in the sensitivity/specificity analysis in which the rTBFmax showed the highest discrimination performance in glioma grading. The estimated effect size for the rTBF was approximately similar between HGGs and LGGs, and grade II and grade III tumours, (–1.46 (–2.00, –0.91), *p*-value < 0.001), (–1.39 (–1.89, –0.89), *p*-value < 0.001), respectively; while it exhibited smaller effect size between grade III and grade IV (–1.05 (–1.82, –0.27)), *p* < 0.05). Sensitivity and specificity analysis replicate these results as well. This meta-analysis suggests that ASL is useful for glioma grading, especially when considering the rTBFmax parameter.

## INTRODUCTION

WHO grade staging of gliomas has implications for prognosis and choice of therapy and MRI plays a leading role in all phases of tumour management, including diagnosis, therapy, and follow-up. T1-weighted post contrast MRI allows identification regions of *blood brain barrier* (BBB) disruption [1, 2] which are usually associated with higher WHO grades but presence of contrast enhancement can be misleading as some low-grade gliomas (LGGs) demonstrate contrast uptake, with lack of enhancement being observed in some high-grade gliomas (HGGs) [3]. Arterial spin labelling (ASL), which uses magnetically labelled blood water as an inherently diffusible tracer, is now performed in clinical settings as, unlike contrast-enhanced perfusion MRI techniques, it can provide absolute cerebral blood flow (CBF) quantification, eliminates the need for contrast agent, and can be repeated for therapy monitoring. Additionally, ASL is less sensitive to vessels permeability changes, which suggests that it provides tumour perfusion information that reflects vascular density [4].

Quantitative ASL measurements have introduced as output relative (or normalised) tumour blood flow (rTBF) and/or absolute tumour blood flow (TBF) values, which have been reported as useful in distinguishing between HGGs and LGGs [5–7] and in glioma grading [8] with some studies reporting nevertheless negative results [9–11]. The purpose of this systematic review and meta-analysis was to address this ambiguity and provide evidence for the diagnostic accuracy of ASL in preoperative glioma grading.

## MATERIALS AND METHODS

### Literature search and selection

**Figure 1:**
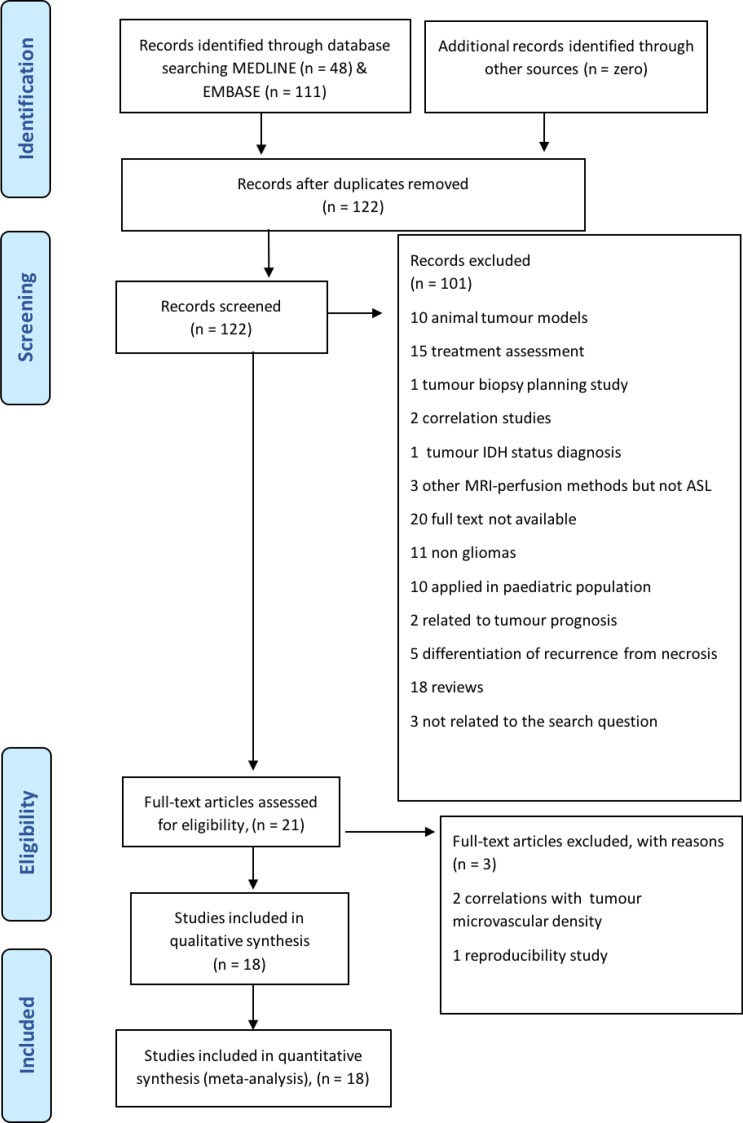
Preferred Reporting Items of Systematic Reviews and Meta-Analyses-PRISMA flow chart for the study selection process

This meta-analysis followed the Preferred Reporting Items for Systematic reviews and Meta-Analyses (PRISMA) guidelines with the research question being ‘What is the diagnostic value of arterial spin labelling (ASL) in the differentiation of glioma grades in adult patients?’ [12]. The search terms were identified according to the Population/Intervention/Comparator/Outcomes (PICO category) recommendations and were linked by Boolean operators (‘OR’ within each PICO category; and ‘AND’ between PICO categories). The identified search terms were framed in concepts. Concept 1 (P): glioma OR neuroglia OR glioma; Concept 2 (I): arterial spin OR artery spin; Concept 3 (O): diagnosis OR grading OR differentiate. Finally, the search was conducted without the third concept in order to cast a wider net. As a result, steps 1 and 2 were combined [(glioma OR neuroglia OR glioma) AND (arterial spin OR artery spin)]. The systematic search was performed in June 2018 through EMBASE ‘(1974 to 8 June 2018)’ and Ovid MEDLINE (R) ‘In-process and other non-indexed citations’ databases to find relevant articles that met the defined search terms; this resulted in the identification of 111 and 48 items respectively. A total of 159 items were found, which were rendered to 122 after duplicates were removed. We included records focusing on pre-treatment glioma grading in adults using ASL. Relevant articles were selected according to PRISMA (see relevant flow chart in Figure 1). 104 of them were excluded as they did not met the research question; for example: they applied ASL in animal models [13] use ASL in order to asses the treatment [14, 15] or applied in pediatric population [16]. Eventually, 18 studies were deemed eligible in terms of the selected inclusion criteria.

Eight out of the 18 studies reported the TBF values as a mean and standard deviation, whereas three reported the cut-off values and the corresponding diagnostic sensitivity and specificity rates. The remaining 7 studies provided all the aforementioned information. Due to high variability in expressing the TBF amongst the studies, we decided to rename the studies output as follows: when the region of interest (ROI) included the entire tumour outlined on conventional images, the absolute or relative to the healthy appearing white matter TBF was referred to as TBFmean/rTBFmean. When the tumour ROI was placed on the highest signal in the perfusion map, the TBF is referred as maximum TBFmax/rTBFmax. In some studies, both mean and maximum TBF/rTBF values were reported.

The considerable variability in the study populations, ASL labelling methods, and acquisition parameters in addition to the diversity of post-processing analyses process among the included studies could potentially limit the power of this study evidence thus the Quality Assessment of Diagnostic Accuracy Studies-2 (QUADAS-2) [17] tool was used by 2 independent researchers to assess the risk of bias of the included studies and their applicability.

### Statistical methods

Analyses were performed for a number of tumour WHO grading combinations: HGGs vs. LGGs; grade II vs. grade III; grade II vs. grade IV; grade III vs. grade IV. All outcomes were measured on a continuous scale. The mean, standard deviation (SD) and number of subjects were extracted from each individual study. For studies where the data range was only reported, the SD was assumed to be a quarter of the whole range. Studies with no reported measure of variability (e.g. SD, inter-quartile range, range) were excluded from this analysis (study 14 and 17 for rTBFmax, still they provided the ROC analysis and TBFmax, respectively). Some studies reported the same parameter from both the whole sample and sub-group (e.g. study 8, Supplementary Table 2); whereas some studies reported the same outcome from the same sample using either different ASL techniques (e.g. study 16, Supplementary Table 2) or different image analysis methodology (e.g. study 18, Supplementary Table 1). In such instances, the different data sets from the same study were treated as being from ‘different’ studies to avoid doubling the study weight. The Chi-square test for heterogeneity was used to determine if the results from different studies varied significantly. Additionally, heterogeneity was quantified using the *I*^2^ statistic, which gives the percentage of the variability in effect estimates that is due to heterogeneity. An *I*^2^ value of over 50% was regarded as indicating substantial heterogeneity.

Subsequently, the results from the different studies were pooled. The measurement scales of some outcomes varied, and thus the standardised mean difference (SMD) between groups was calculated in preference to the raw mean difference. A random-effects model was used for all meta-analyses, regardless of the degree of heterogeneity between studies. The examination of publication bias was investigated using graphical methods (Funnel plots) examining the association between the effect size (SMD) and the uncertainty (standard error) in the calculating effect size.

The diagnostic performance (sensitivity and specificity) of the ASL in differentiating between HGGs and LGGs and in glioma grading were also investigated. The data analysis used an approach outlined by Li et al. [18, 19] that jointly models the two outcomes (sensitivity and specificity), due to the known inverse relationship between these two measures. The approach taken here was to fit a two-level mixed logistic regression model, with independent binomial distributions for the true positives and true negatives conditional on the sensitivity and specificity in each study, and a bivariate normal model for the logit transforms of sensitivity and specificity between studies. This approach gives pooled estimates of sensitivity and specificity, along with corresponding confidence intervals (CI) for each. A hierarchical summary receiver operating characteristic (HROC) curve was also generated. This analysis was performed using the “*metandi”* command in STATA 15 (StataCorp LLC, College Station, TX, USA).

## RESULTS

### Eligible ASL studies

The studies are grouped according to the applied ASL acquisition technique - pseudo-continuous-ASL, continuous-ASL and pulsed-ASL (PCASL, CASL, PASL, respectively) - and summarised on Supplementary Tables 1–3. The examined gliomas histology, the ASL technical parameters, the selected TBF metrics, and any statistical significant difference between HGGs and LGGs are also presented on Supplementary Tables 1–3.

### QUADAS-2 assessment

**Figure 2:**
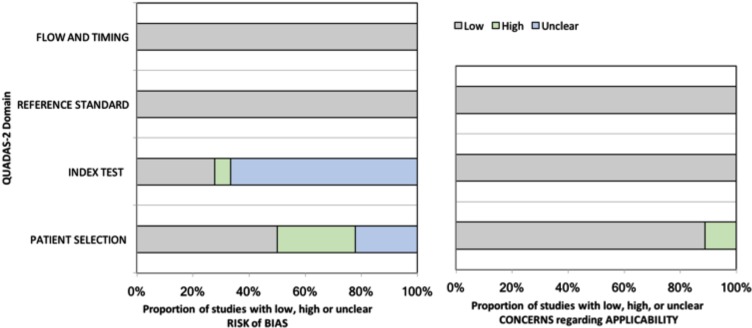
The Quality Assessment of Diagnostic Accuracy Studies-2 (QUADAS-2) results for the included studies

Five of the included studies expressed low risk of bias and concerns regarding applicability following the QUADAS-2 assessment. The summary graph of QUADAS-2 assessment is demonstrated in Figure 2. The risk of bias graph shows the four domains; the flow and timing and the reference standard domains possess the lowest risk of bias. The index test domain, could introduce bias as in the majority of the included studies the reviewers were not blinded to the standard reference (high) or the authors did not provide this information. In the patient selection domain, about 25% of the eligible for meta-analysis studies did not use a consecutive or random selection (high) while ~ 25% did not explain the process of patient selection (unclear). The concerns regarding applicability graph involved three domains; the reference standard and the index test domains showed low concerns as all the studies included ASL as index test and histopathological examination as a reference standard. However, the patient selection domain expressed ~10% (high) as one of the studies included both adult and pediatric patients and another one include residual gliomas in their analysis.

### Differentiation of HGGs from LGGs

With the exception of TBFmean, there was considerable heterogeneity among the studies (Table 1). The Chi-square test for heterogeneity was statistically significant, and additionally the *I*^2^ values were high. All ASL-derived biomarkers were found to be significantly lower in LGGs than in HGGs. The larger effects were observed for the rTBF, where the sizes of group differences were typically higher than for the TBF. The mean differences between LGGs and HGGs for the rTBF was approximately 1.5 SDs. The rTBF funnel plots typically showed values outside the confidence limit in both directions, reflecting the large study heterogeneity. However, there was no clear picture that the effect size was associated with the standard error of the SMD to suggest publication bias (Egger test, *P*-value = 0.17, 0.72, 0.24 for rTBF, rTBFmean and rTBFmax, respectively). The TBF funnel-plots suggested that the majority of points were within the confidence limits, corroborating the lack of obviouas publication bias (Egger test, *P*-value = 0.05, 0.43, 0.10 for TBF, TBFmean and TBFmax, respectively). A graphical illustration of the results is shown in the Supplementary Figures 1–6.

**Table 1:** Comparison of the differences in ASL-related biomarkers between HGGs and LGGs and between different glioma grades

Biomarkers	Number of studies	Total sample size		Heterogeneity		Effect size		Egger test to evaluate publication bias
		LGG	HGG	*P*-value	*I*^2^	SMD (95% CI) (^*^)	*P*-value	*P*-value
rTBF	15	237	323	<0.001	86%	–1.46 (–2.00, –0.91)	<0.001	0.17
rTBF mean	9	142	192	<0.001	86%	–1.53 (–2.26, –0.79)	<0.001	0.72
rTBF max	6	95	131	<0.001	87%	–1.36 (–2.23, –0.49)	0.002	0.24
TBF	11	155	219	0.002	64%	–0.82 (–1.20, –0.45)	<0.001	0.05
TBF mean	4	51	70	0.50	0%	–0.61 (–0.99, –0.23)	0.002	0.43
TBF max	7	104	149	<0.001	76%	–0.96 (–1.53, –0.39)	0.001	0.10
		**II**	**III**					
rTBF	4	62	48	0.26	25%	–1.39 (–1.89, –0.89)	<0.001	0.46
TBF	2	43	21	0.09	66%	–0.90 (–1.85, 0.04)	0.06	(-)
		**II**	**IV**					
rTBF	4	62	61	<0.001	87%	–2.07 (–3.38, –0.76)	0.002	0.25
TBF	2	43	32	0.01	84%	–1.44 (–2.76, –0.12)	0.03	(-)
		**III**	**IV**					
rTBF	6	54	69	0.006	69%	–1.05 (–1.82, –0.27)	0.008	0.19
TBF	4	27	40	0.64	0%	–0.45 (–0.95, 0.05)	0.08	0.04

(^*^) SMD calculated as the difference between LGGs ASL-parameters and HGGs ASL-parameters (usually higher than the LGG counterparts).

(-) No pooled results due to low number of included studies.

### Differentiation of grade II from grade III gliomas

A summary of the analysis results is presented on Table 1. rTBF values were characterised by relatively small degree of heterogeneity between studies and were found to significantly vary between the two investigated WHO grades, with substantially lower values in grade II patients. The mean rTBF value was 1.4 SDs lower in grade II than in grade III patients. There were only two studies reporting absolute TBF values, and there was a relatively large amount of heterogeneity in them (*I*^2^:66%). The absolute TBF showed a trend for lower in grade II patients by an average of 0.9 SDs. Concerning publication bias, all points were within the confidence limits and there was no clear evidence of asymmetry of the funnel plot (Egger test, *P*-value = 0.46 for rTBF). Graphical illustrations of these results are shown in Supplementary Figures 7 and 8.

### Differentiation of grade II from grade IV gliomas

The ASL-derived parameters between grade II and grade IV patients are summarised on Table 1. The results showed significant heterogeneity among the included studies with ASL biomarkers showing significantly lower values in grade II patients. The larger effect was observed for rTBF, which was 2 SDs lower in grade II patients than in grade IV gliomas. The effect size for TBF was substantial (1.4 SDs). The funnel-plot for the rTBF was fairly symmetrical suggesting no definite evidence of publication bias (Egger test, *P*-value = 0.25 for rTBF). The results are illustrated graphically in Supplementary Figures 9 and 10.

### Differentiation of grade III from grade IV gliomas

The differences in ASL-derived tumour perfusion between grade III and grade IV gliomas are summarised on Table 1 and suggest a significant degree of heterogeneity (*I*^2^:69%) through the 6 studies that provided rTBF as ASL output. The pooled results suggest significantly lower rTBF values in grade III patients compared to their grade IV counterparts. The SMD between grades was 1 SD, which is slightly lower than the difference between grade II and III gliomas. The results for TBF showed little heterogeneity between studies and a trend for lower TBF in the grade III patients (*p* = 0.08). The funnel plot appeared fairly asymmetrical for TBF, with obviously larger SMD values being found in the smaller studies (owing to larger standard errors) and smaller effects in the larger patient cohorts (with smaller SE) but not for the rTBF (Egger test, *P*-value = 0.04, 0.19 for TBF and rTBF, respectively). For both ASL-derived parameters, there was some suggestion of publication bias (see Supplementary Figures 11 and 12).

### HROC curve analysis of ASL-based histological grading

**Figure 3:**
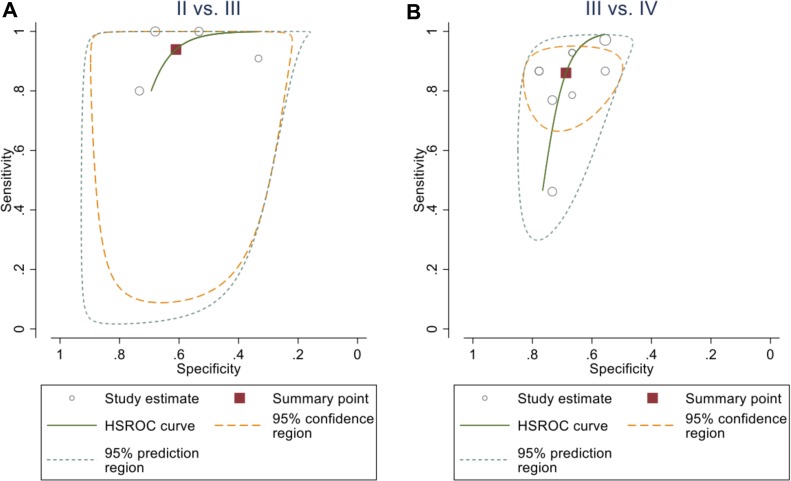
HROC plot of the summary point of the sensitivity and the specificity (square) and its 95% CI (the green curve) of rTBF from ASL to differentiate between (**A**) grade II and grade III (94%, CI (75%, 99%)) and (61%, CI (48%, 73%)), respectively; (**B**) grade III and grade IV (86%, CI (75%, 93%)) and (69%, CI (57%, 79%)), respectively.

The diagnosic performance of ASL for determining the individual glioma grades (Table 2) was characterised by high sensitivity (>90%) for diffrentiation between grade II and III. The sensitivity was slightly lower for the classification between grade III and IV, but still relatively high. This is in agreement with the aforementioned results as the effect size between grade II and grade III gliomas was higher than that between the grade III and grade IV tumours. The method specificity for the individual tumour grading was below 70%. Both the 95% confidence and prediction regions indicate wide variability of the true sensitivity and specificity. The ASL sensitivity, specificity, negative predictive value (NPV) and positive predictive value (PPV) for various cut-off values in glioma grading from the involved studies illustrated on Supplementary Table 4. The HROC plots are showed on Figure 3.

**Table 2:** Diagnostic performance of the ASL in discrimination between glioma grades

Glioma grading	Number of studies included	Total sample size		Sensitivity (95% CI)	Specificity (95% CI)	AUC (95% CI)
II vs. III	4	52	64	94% (75%, 99%)	61% (48%, 73%)	0.76, (0.72, 0.79)
II vs. IV	3	(^*^)	(^*^)	(^*^)	(^*^)	(^*^)
III vs IV	9	148	75	86% (75%, 93%)	69% (57%, 79%)	0.75, (0.71, 0.79)

(^*^) No pooled results due to low number of included studies.

### HROC curve analysis of ASL-based differentiation between HGGs and LGGs

**Figure 4:**
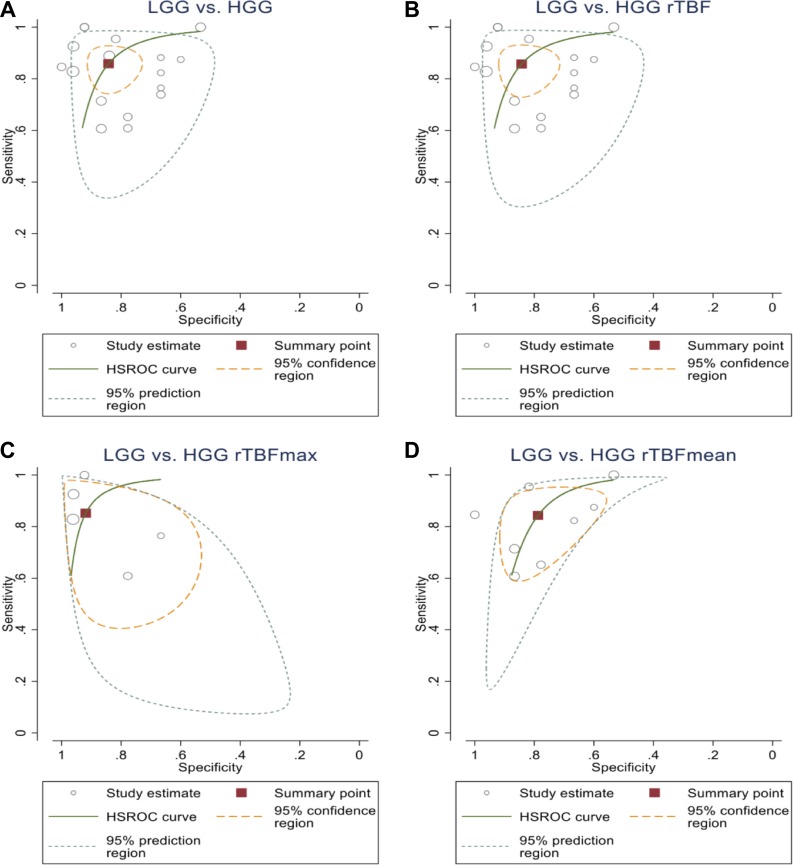
HROC plot shows the summary point of the sensitivity and the specificity (square) and its 95% CI (the green curve) (**A**) from all of the analysed ASL parameters to differentiate between HGGs and LGGs (86%, CI (78%, 91%)) and (84%, CI (76%, 90%)), respectively; (**B**) rTBF to differentiate between HGGs and LGGs (86%, CI (77%, 91%)) and (84%, CI (76%, 90%)), respectively; (**C**) rTBFmean to differentiate between HGGs and LGGs (84%, CI (71%, 92%)) and (79%, CI (66%, 88%), respectively; (**D**) rTBFmax to differentiate between HGGs and LGGs (85%, CI (69%, 94%)) and (92%, CI (80%, 97%)).

The overall results suggested relatively high sensitivity and specificity, both approx. 85%, by using ASL biomarkers to stratify the gliomas in HGGs and LGGs. There was a similar level of sensitivity for each of the individual ASL parameters under analysis. Specificity was slightly more variable ranging from 79% for rTBF mean up to 92% for rTBF max. Both the 95% confidence and prediction regions indicate wide variability of the true sensitivity and specificity. A summary of the results is presented on Table 3. The sensitivity, specificity, negative predictive value (NPV) and positive predictive value (PPV) of various cut-off ASL values among glioma grades from the involved studies are shown in Supplementary Table 5 with the HROC plots presented in Figure 4.

**Table 3:** Diagnostic performance of the ASL imaging biomarkers in stratifying the tumours between HGGs and LGGs

Biomarker	Number of studies	Total sample size		Sensitivity (95% CI)	Specificity (95% CI)	AUC (95% CI)
		LGGs	HGGs			
All	17	206	397	86% (78%, 91%)	84% (76%, 90%)	0.91, (0.89, 0.93)
TBF	1	(^*^)	(^*^)	(^*^)	(^*^)	(^*^)
rTBF	16	181	370	86% (77%, 91%)	84% (76%, 90%)	0.91, (0.89, 0.94)
rTBF max	5	76	122	85% (69%, 94%)	92% (80%, 97%)	0.95, (0.93, 0.97)
rTBF mean	8	80	188	84% (71%, 92%)	79% (66%, 88%)	0.87, (0.84, 0.90)

(^*^) No pooled results due to low number of included studies.

## DISCUSSION

The results of this meta-analysis indicate that the absolute TBF can be used to differentiate HGGs from LGGs as well as grade II from grade IV gliomas. However, absolute TBF could not discriminate grade II from grade III and grade III from grade IV glial tumours. In contrast, rTBF was more effective than absolute TBF in differentiating HGGs from LGGs and presented satisfactory accuracy in glioma grading. In addition, rTBFmax parameter demonstrated the best performance in glioma grading overall. The same result was observed in the sensitivity and specificity analysis where the rTBFmax provided the highest sensitivity and specificity values. The estimated effect size for rTBF was approximately similar between HGGs and LGGs (−1.46 (−2.00, −0.91), *p*-value < 0.001), and between grade II and grade III (−1.39 (−1.89, −0.89), *p*-value < 0.001), while it expressed smaller effect size between grade III and grade IV (−1.05 (−1.82, −0.27)), *p* < 0.05). The same result was also noticed in the sensitivity and specificity analyses. Fudaba et al. also reported that rTBFmax provided higher sensitivity and specificity than rTBFmean [20].

The systematic literature review and the heterogeneity analysis highlighted the variations across the included studies, which could be due to inclusion of mixed glioma types, the applied ASL approaches and its parameters (e.g post-labelling-delays (PLD)), and the method of image processing and analysis. Notably, all studies that reported the inability of ASL to distinguish HGGs from LGGs [9–11] were conducted in mixed gliomas patient cohorts. This sounds rational as even low-grade oligodendrogliomas are associated with elevated perfusion and thus introduce diagnostic bias [7, 21]. A study that included only oligodendrogliomas found also impossible to determine the WHO grade using ASL biomarkers [22]. On contrary, a handful of studies in astrocytomas demonstrated that ASL can identify HGGs from LGGs [23–25] and grade them accordingly with remarkable sensitivity and specificifity [8, 20]. In spite of that, other studies with mixed glioma types reported the efficiency of ASL in differentiating between HGGs and LGGs [6, 7] and glioma grading [5]. The 2016 WHO classification of brain tumours puts more emphasis on the genetic and molecular subtyping of gliomas by stratifying them according to the isocitrate-dehydrogenase-(IDH) and 1p/19q mutation status or co-deletion, respectively [26]. The ASL studies reviewed in this meta-analysis lacked information about molecular and genetic subtypes of gliomas, which is likely to represent a source of variation as the IDH-wild gliomas has been reported to have higher perfusion values than those with IDH-mutation [9, 27].

The different ASL labelling approaches and acquisition parameters across the included studies obviously result in quantitative ASL metrics variation. The three common ASL labelling methods used in these studies were the pulsed-ASL (PASL); the continuous-ASL (CASL); and the pseudo-continuous-ASL (PCASL). PASL is the most widely used technique due to its broad availability, the low specific absorption rate (SAR), and the robust labelling efficiency [28] across a wide range of blood velocity. However, this method suffers from the lowest signal-to-noise-ratio (SNR) in comparison to the other labelling methods. CASL is subject to higher SAR and lower labelling efficiency than PASL. PCASL has the advantages of both CASL (high SNR) and PASL (low SAR) however, its labelling efficiency is lower than that of PASL. Thus, PCASL has been reported to demonstrate the best reproducibility among all aforementioned ASL labelling methods, at least in healthy volunteers [29].

All but two studies with negative results [9, 30] that applied PASL in gliomas showed its efficiency in differentiating between HGGs and LGGs [7, 20, 23–25, 31, 32] and in glioma grading [8]. All studies that utilised CASL in gliomas [21, 22, 30] were promising in differentiation between HGGs and LGGs. Nonetheless, recent studies drew conflicting conclusions regarding the usefulness of PCASL acquisitions as it helped to identify HGGs from LGGs [6] and to perform grading [5], whilst a couple of publications didn't confirm the findings [9–11]. This discrepancy could be due to the low labelling efficiency of PCASL, which varies between scanners and patients. Another important acquisition parameter in ASL is the inversion time (TI), also called post labelling delay (PLD). Selection of the most suitable TI in tumours tends to be challenging as ASL acquisition at a single TI reduces the sensitivity to the blood transit time but does not eliminate it. This sensitivity can be considered by the use of multiple PLDs [33] in CASL/PCASL or by quantitative-image of perfusion using a single-subtraction-(QUIPSS-II) [34]/QUIPSS-II with thin slice TI1 periodic saturation (Q2TIPS) [35] in PASL. Generally, the delay time has to be long enough to enable all the labelled blood bolus to transfer from the capillary bed to the target tissue in the labelling plane, but short enough to preserve the signals from T1-decay. Furtner et al. identified the most effective TI for determining the HGGs from the LGGs astrocytomas at 370 ms [23]. Other studies employed variable single delay times within a range of 1200–1900 ms, and reported similar or lower sensitivity and specificity [6, 8, 20, 24, 30, 31]. Unlike using a single TI, the use of multiple TIs makes CBF quantification more reliable and less sensitive to bolus arrival time. Cebeci et al. used 8 TIs to distinguish HGG from LGG [7] and Yang et al. acquired ASL at 16 TIs in astrocytomas, which not only differentiated HGGs from LGGs, but also enabled glioma grading [8].

The reported tumour perfusion was heavily skewed by the non-standardised ROI analysis on the calculated CBF maps. Normalised TBF values (also known as rTBF) have been widely used as they reduce the values scatterring within a group by mitigating age and hemodynamic variations related risks [25]. Also, patients with brain tumours usually present elevated intracranial pressure, which in turn reduces the global CBF and the accuracy of the estimation of regional TBF. On the other hand, several authors argue that the use of rTBF leads to variation between observers due to random error caused by the internal reference on the normal tissue [36, 37]. This could partially explain the high variation in the rTBF of malignant tumours. Nevertheless, the relative values are considered more reliable than the absolute TBF values when distinguishing between HGGs and LGGs [7, 25] which has also been confirmed by our meta-analysis. Previous ASL tumour studies have used various normal brain regions for normalisation, including the GM [38] the WM [31], the mean of both [32], or the contralateral normal tissue mirrored to tumour [25]. Interestingly, the mirror ROI yielded better results than GM and WM as an internal standard [39], which is most likely due to the approximately similar distance from the labelling plane. Nevertheless, the majority of studies have used the contralateral normal appearing white matter for normalisation purposes [5, 8, 20, 22, 24, 31, 40]. Furthermore, it is important to note that the image employed to delineate the tumour mask may affect the reliability and validity of results. A number of studies generated tumour mask from the MRI-conventional image whilst others selected the ROI through visual inspection of the maximum signal intensity visually on the ASL-subtracted image or the M0 image. Regarding the choice of contrast-enhanced T1-weighted images as reference for tumour mask, we should bear in mind that the enhanced tissue represents blood brain barrier disruption (BBB) rather than increased perfusion [41].

In line with our meta-analysis results, TBFmax has been reported to be more precise than TBFmean for tumour characterisation [42, 43]. This is plausible, as HGGs tend to be heterogeneous, and hence the TBFmax will be representative of the most anaplastic tumour part. In addition, TBFmean estimation is affected by the partial volume effect making the TBFmax more suitable as a biomarker. Nevertheless, histogram analysis [44, 45] that also captures the tumour heterogeneity is probably the method with the highest diagnostic accuracy and reproducibility.

Several studies have examined ASL feasibility and its complementary role in routine brain tumour examinations by comparing it with more well-established MRI methods; to other MRI-perfusion methods, dynamic susceptibility contrast-enhanced (DSC) [7, 25, 43] and dynamic contrast-enhanced (DCE) [9, 10] to MRI-diffusion methods (MRI diffusion) [5, 6, 31] and to MR-spectroscopy (MRS) [20, 22, 32]. Currently, dynamic susceptibility contrast (DSC) is the clinically most utilised MR perfusion technique in brain tumour examinations. However, arterial spin labelling (ASL), is useful for those who could not tolerate high-rate contrast injection or (relatively) contraindicated to the usage of contrast agent (impaired renal function, allergies, paediatric population) along with raising issues over the permanent gadolinium depositions in brain [46]. The studies that involved both indexed examinations in glioma grading, focused on the use of ASL as an alternative or surrogate of DSC [7, 25, 37] e.g. by examining the correlation or interchangeability of the estimated perfusion metrics from each examination, rather than suggesting which method is superior. As a matter of fact, these studies demonstrate the non-inferiority of ASL in the specific patient cohorts. Warmuth et al. reported a strong positive correlation between the rTBF measurement from ASL and DSC [25]. Another study measured rTBFmax and rTBFmean reporting excellent correlation between ASL and DSC [37]. Cebeci et al. used PASL to demonstrate moderate but still significant correlation between the rTBF values from the ASL and DSC [7]. These studies provide preliminary evidence that ASL can be used as a non-invasive alternative to DSC addressing the shortcoming of non-diffusible tracer (gadolinium) in DSC that leaks out of dysfunctional BBB and may lead to underestimation of the rTBF measurement [47]. In addition, there is scarce evidence on the prognostic role of each perfusion techniques [48–53], where rCBV measurements seem to provide the best sensitivity and specificity to predict tumor recurrence and survival time in gliomas patients [54].

Two of the included studies in this meta-analysis examined the correlation between ASL and DCE in gliomas [9, 10]. Both studies (utilising PASL and PCASL) suggested that ASL was not an effective method for glioma grading and reported poor to moderate correlation between ASL and DCE. This might be attributed to a variation in ROI selection and number of high-grade gliomas in each study. In order to consider ASL as a viable alternative to other MRI perfusion methods (DSC and DCE), its reproducibility and inter-observer variability in patients with brain tumours have to be tested. A number of studies have reported good inter-observer variability in tumour patients using PASL [8, 24] quantitative STAR labeling of arterial regions (QUASAR) [42] and PCASL [5, 11]. Hirai et al. reported excellent reproducibility in glioma patients using both maximum and mean TBF [42].

Regarding the future directions of ASL imaging in tumours, Yoo et al. recenty used PCASL to investigate the connection between the HGGs perfusion values and genetic biomarkers [55] and found the epidermal growth factor receptor (EGFR) to be significantly correlated with rTBF and absolute TBF. Furthermore, Yamashita et al. demonstrated that TBF and rTBF values were significantly greater in GBM patients with IDH-wild type status than those with IDH-mutation [27] and Brendle et al. reported that ASL, unlike DCE, could stratify astrocytomas accortding to the IDH-mutation status [9].

There are some limitations in our study. First, approximately all the evaluated perfusion metrics founde to be heterogenous among the included studies in this meta-analysis. This is expected, because heterogeneity among MRI measurments are unavoidable as they differ from center to center and even between platformas in the same center. However, this heterogeneity has been taken into account in the analysis stage via random effect model utilization. Second, the small sample size of the included studies (18 studies). Still, this reflect the strict methodological standard in order to be faithfull to the assigned research question.

## CONCLUSIONS

This meta-analysis aimed to shed light into the diagnostic performance of ASL in glioma grading and demonstrated the suitability of ASL-derived perfusion metrics in glioma grading. rTBFmax showed the best diagnostic and staging performance. Hence, ASL metrics capacity as imaging biomarkers can be routinely useful for the characterisation and staging of gliomas at baseline, with possible implications for treatment selection and surveillance imaging. However, further research with larger numbers of patients and well defined tumour subtypes, including molecular information, is needed to refine any TBF-relate threshold values that allow higher diagnostic and prognostic accuracy and are essential for the wide dissmination of the technique.

## SUPPLEMENTARY MATERIALS FIGURES AND TABLES


