# Supplementary material for: The value of arterial spin labelling in adults glioma grading: systematic review and meta-analysis
Source: Oncotarget. 2019 Feb 22;10(16):1589–601. doi: 10.18632/oncotarget.26674 (PMC6422184; doi:10.18632/oncotarget.26674)
Supplement: Supplementary file 1 [file oncotarget-10-1589-s001.pdf]

The value of arterial spin labelling in adults glioma grading: systematic review and meta-analysis

SUPPLEMENTARY MATERIALS

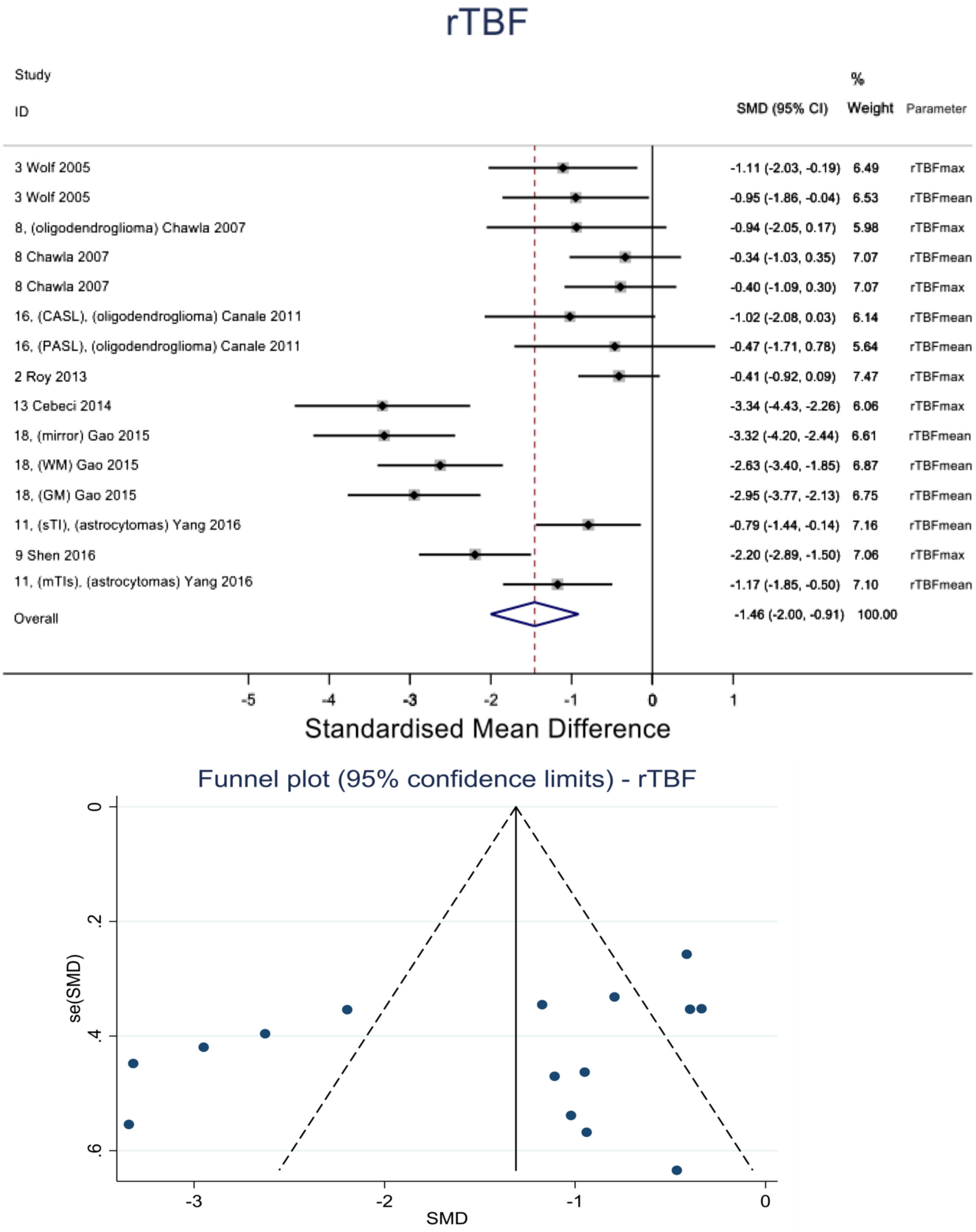

Supplementary Figure 1: rTBF for LGG patients' relative to the value for HGG patients'. In the forest plot, the dotted vertical line represents the pooled effect size point where the effect size in individual studies have very different distribution (heterogeneity) around this line. The pooled effect and their 95% CI (the diamond at the bottom) express that the LGG have significantly lower rTBF than the HGG (–1.46, (–2.00, –0.91)). The funnel plot is symmetric and does not show publication bias.

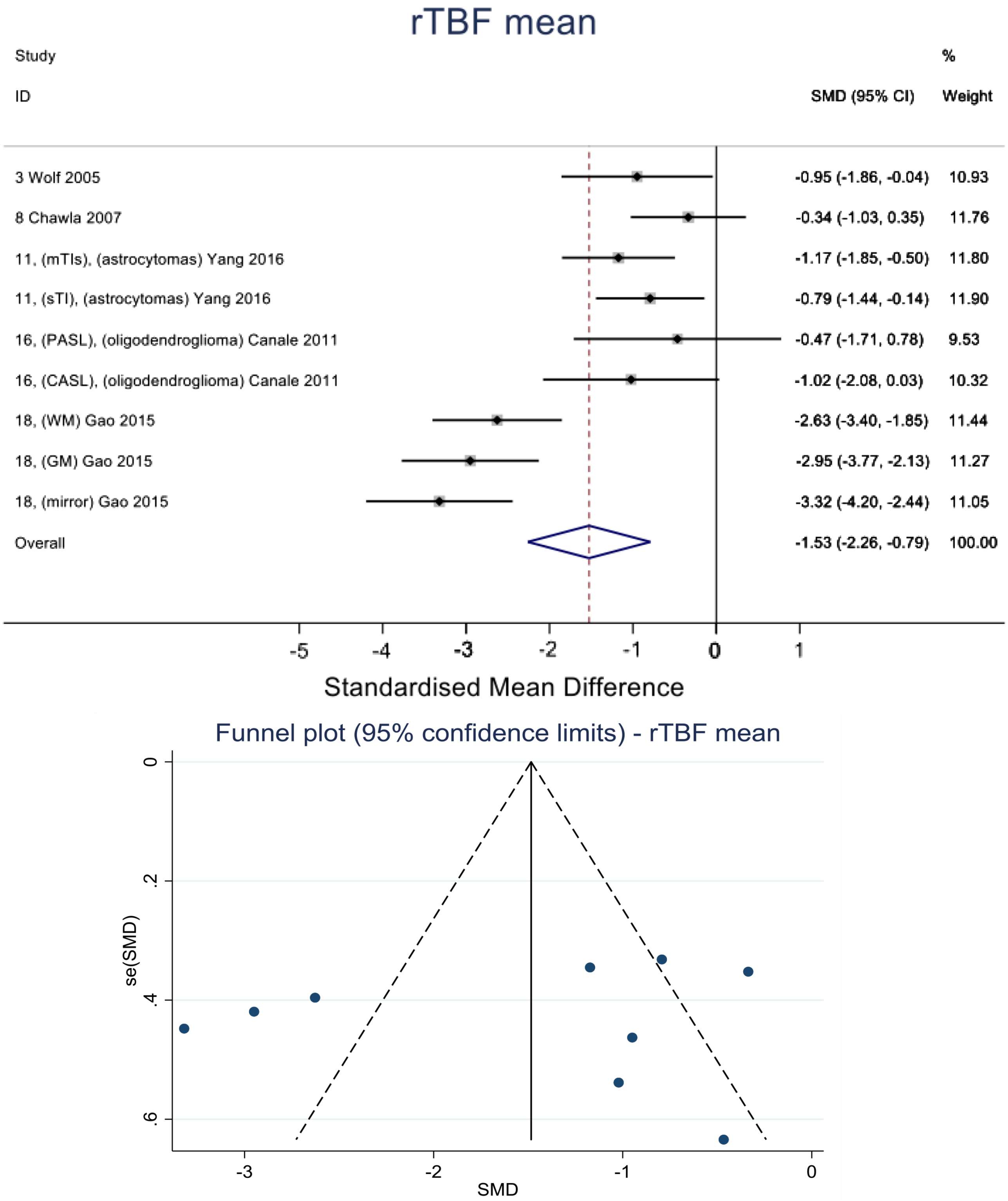

Supplementary Figure 2: rTBF-mean for LGG patients relative to the value for HGG patients. In the forest plot, the dotted vertical line represents the pooled effect size point where the effect size in individual studies have very different distribution (heterogeneity) around this line. The pooled effect and their 95% CI (the diamond at the bottom) express that the LGG have significantly lower rTBFmean than the HGG (–1.53, (–2.26, –0.79)). The funnel plot is symmetric and does not show publication bias.

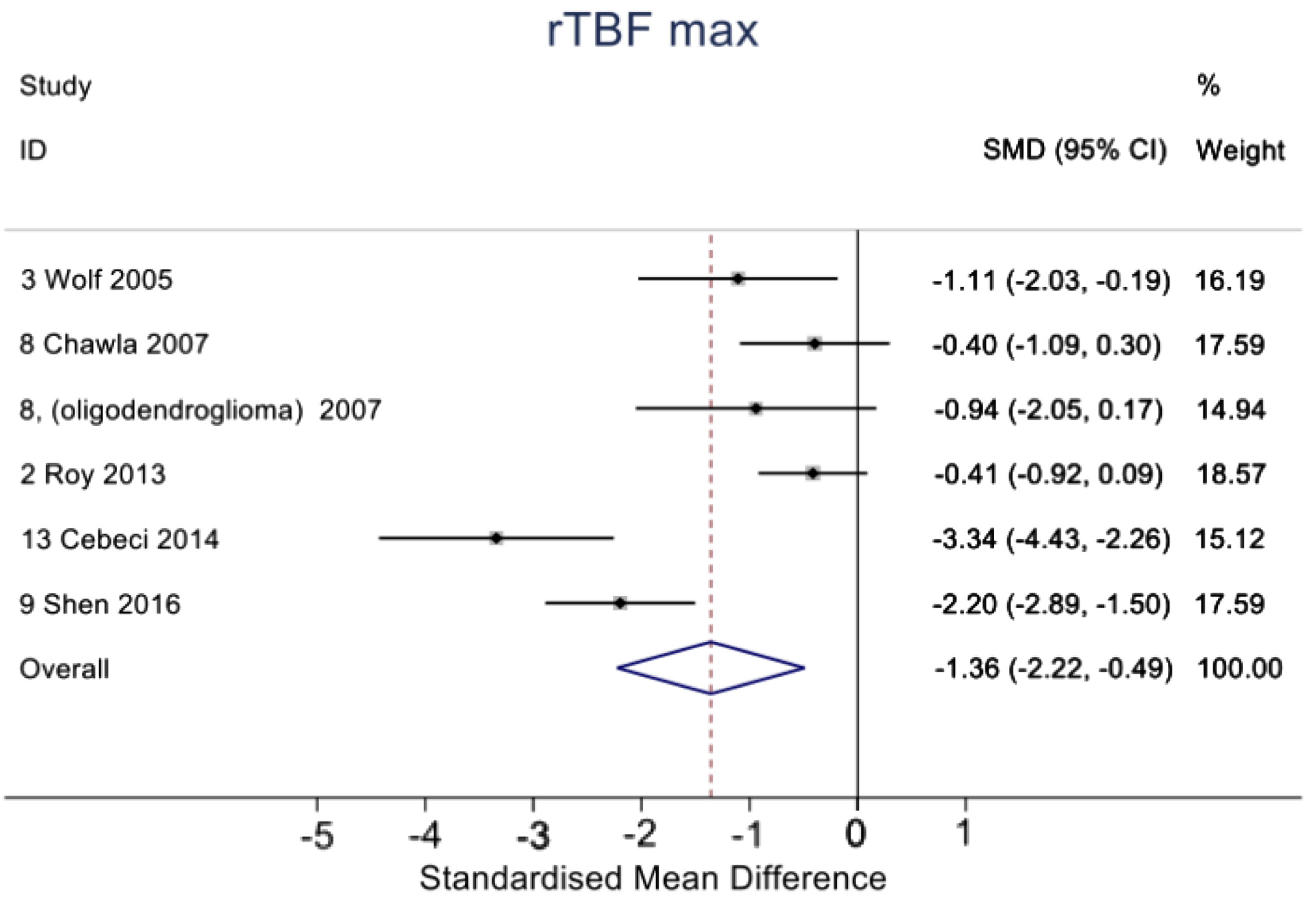

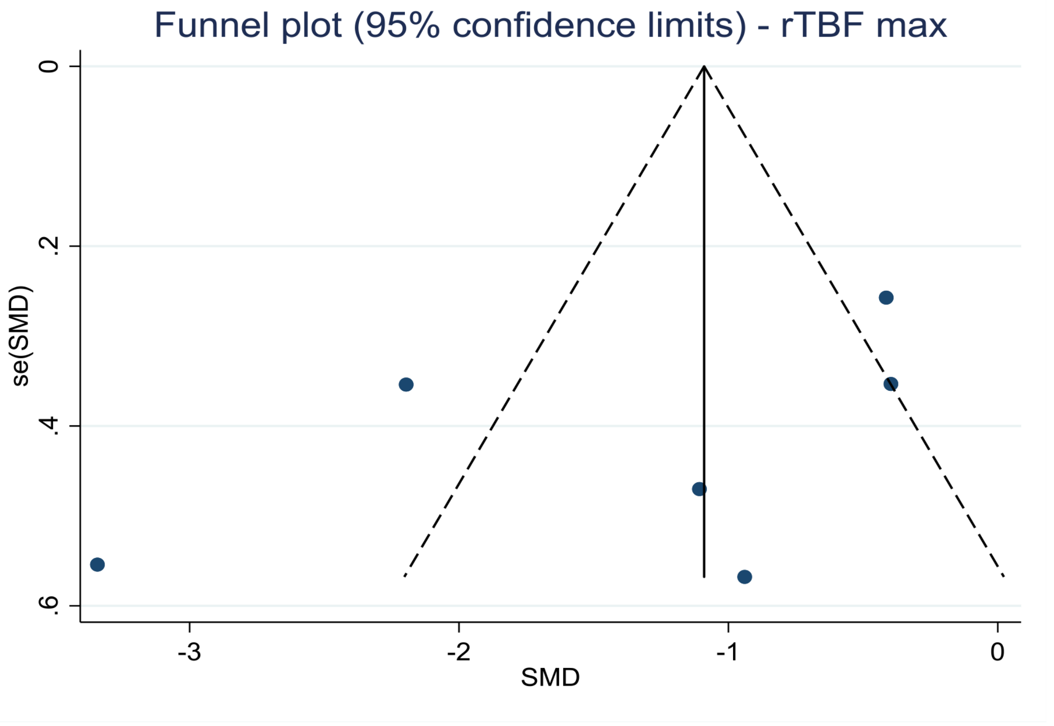

Supplementary Figure 3: rTBF-max for LGG patients' relative to the value for HGG patients'. In the forest plot, the dotted vertical line represents the pooled effect size point where the effect size in individual studies have very different distribution (heterogeneity) around this line. The pooled effect and their 95% CI (the diamond at the bottom) express that the LGG have significantly lower rTBFmax than the HGG (–1.36, (–2.23, –0.49)). The funnel plot is symmetric and does not show publication bias.

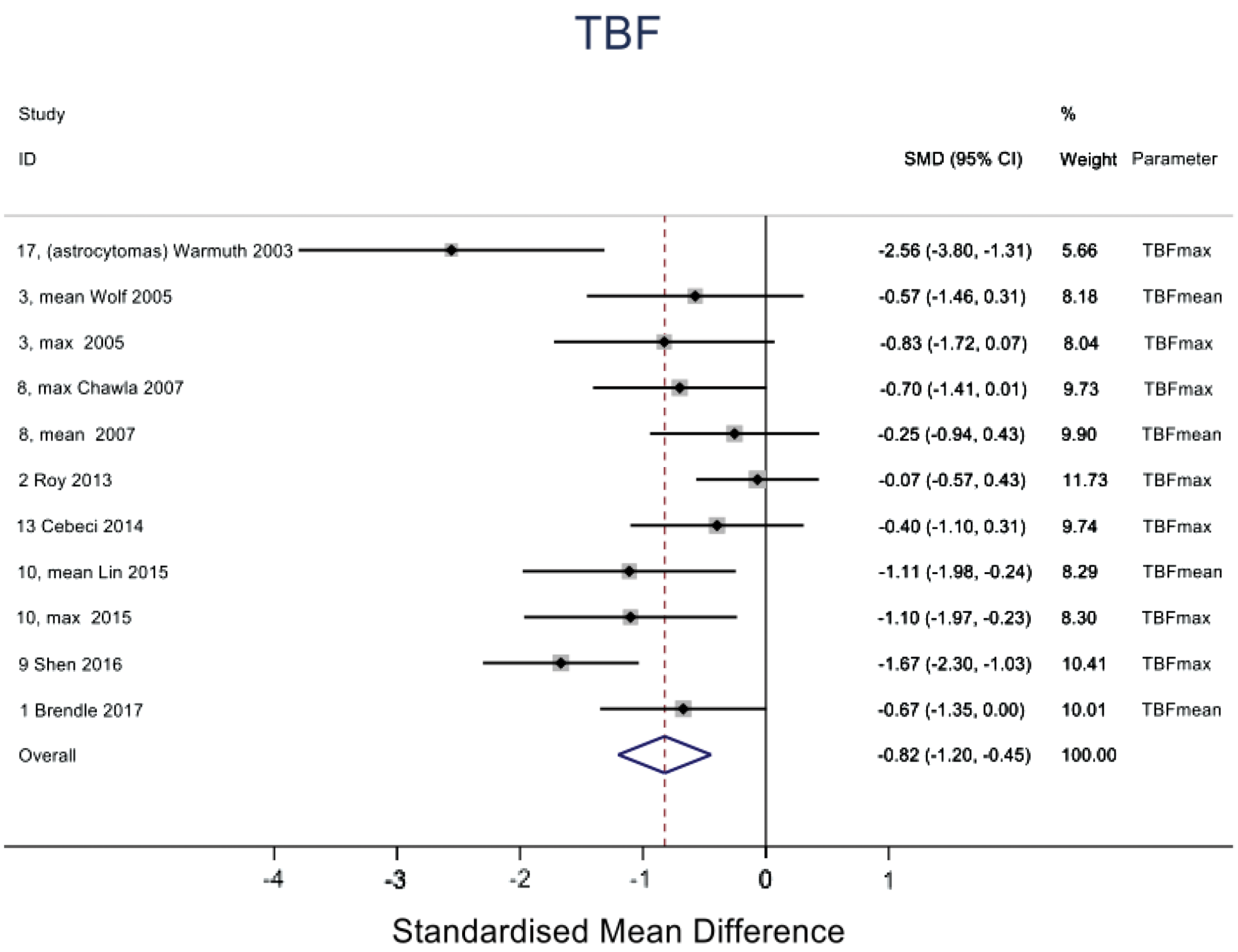

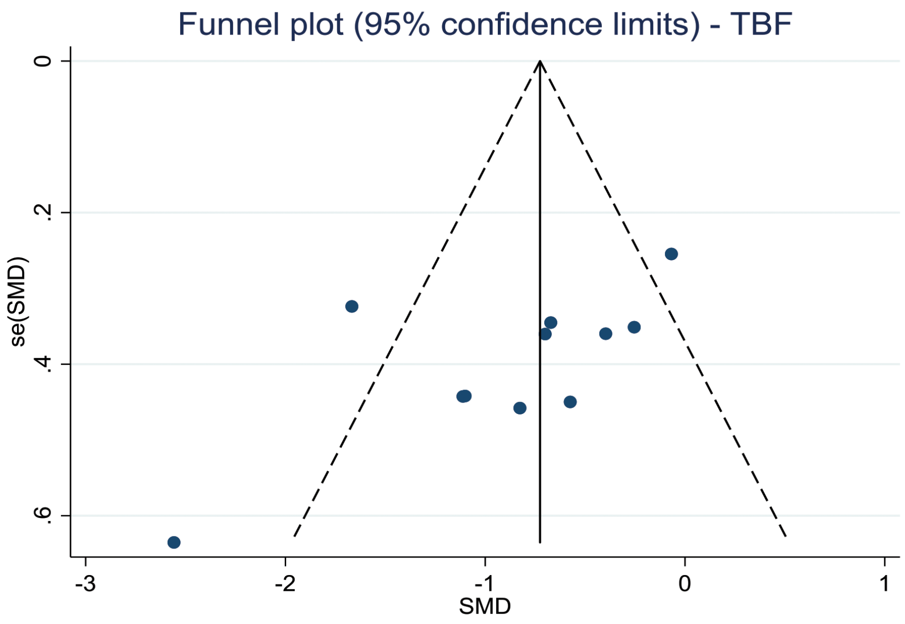

Supplementary Figure 4: TBF for LGG patients' relative to the value for HGG patients'. In the forest plot, the dotted vertical line represents the pooled effect size point where the effect size in individual studies have very different distribution (heterogeneity) around this line. The pooled effect and their 95% CI (the diamond at the bottom) express that the LGG have significantly lower TBF than the HGG (–0.82, (–1.20, –0.45)). The funnel plot is symmetric and does not show publication bias.

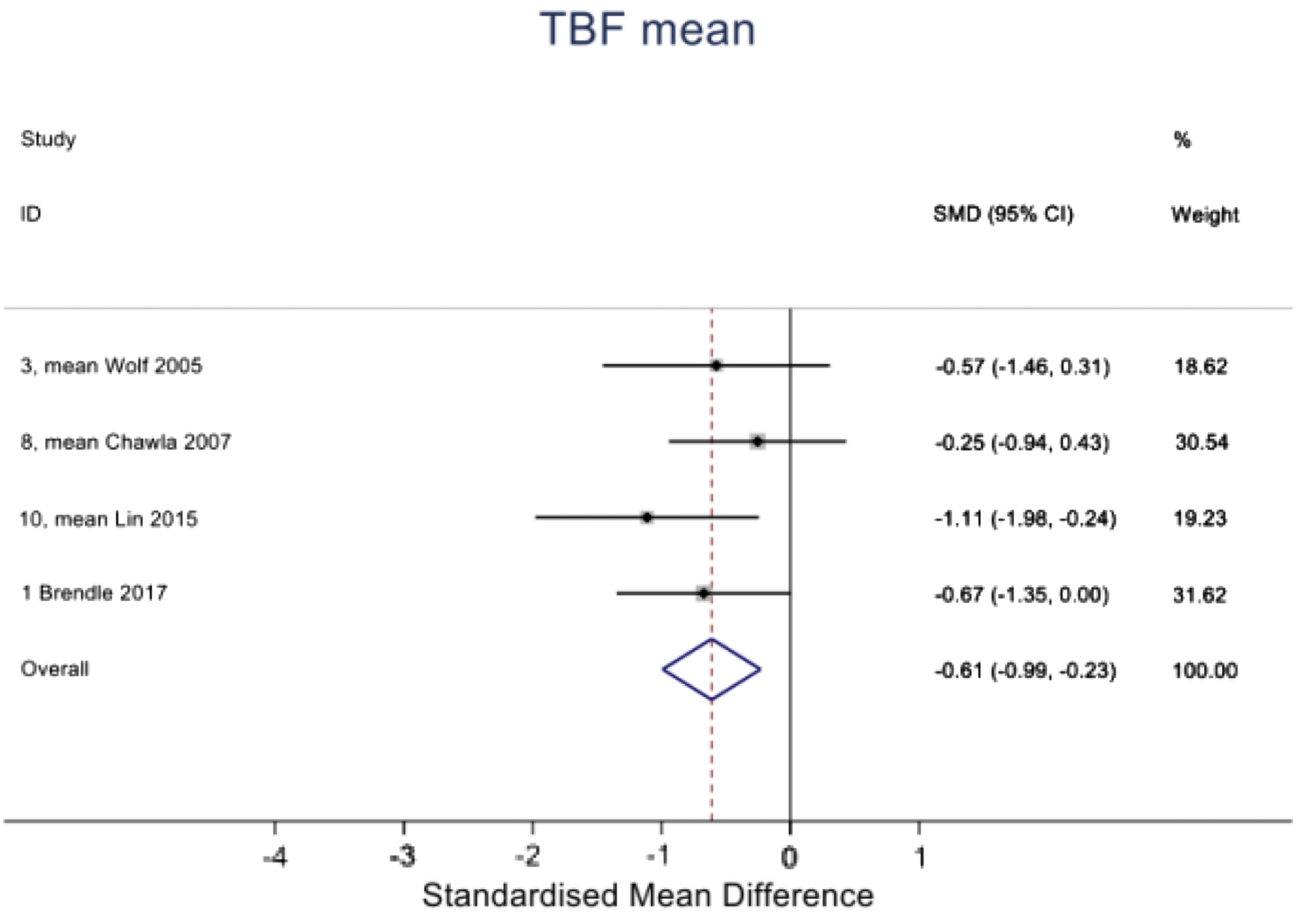

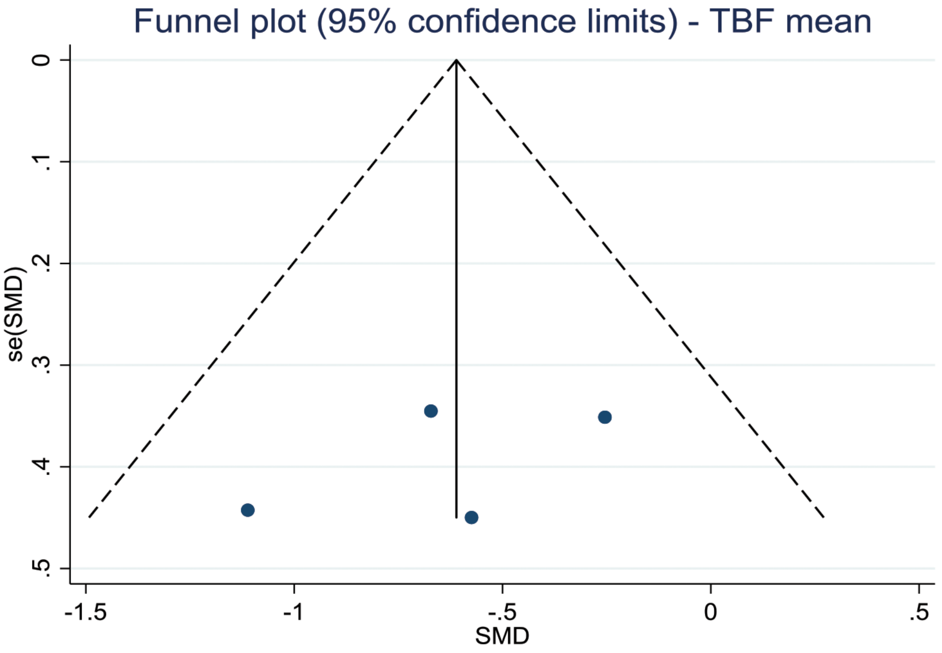

Supplementary Figure 5: TBFmean for LGG patients' relative to the value for HGG patients'. In the forest plot, the dotted vertical line represents the pooled effect size point where the effect size in individual studies have small distribution around this line with small degree of heterogeneity. The pooled effect and their 95% CI (the diamond at the bottom) express that the LGG have significantly lower TBFmean than the HGG (–0.61, (–0.99, –0.23)). The funnel plot is symmetric and does not show publication bias.

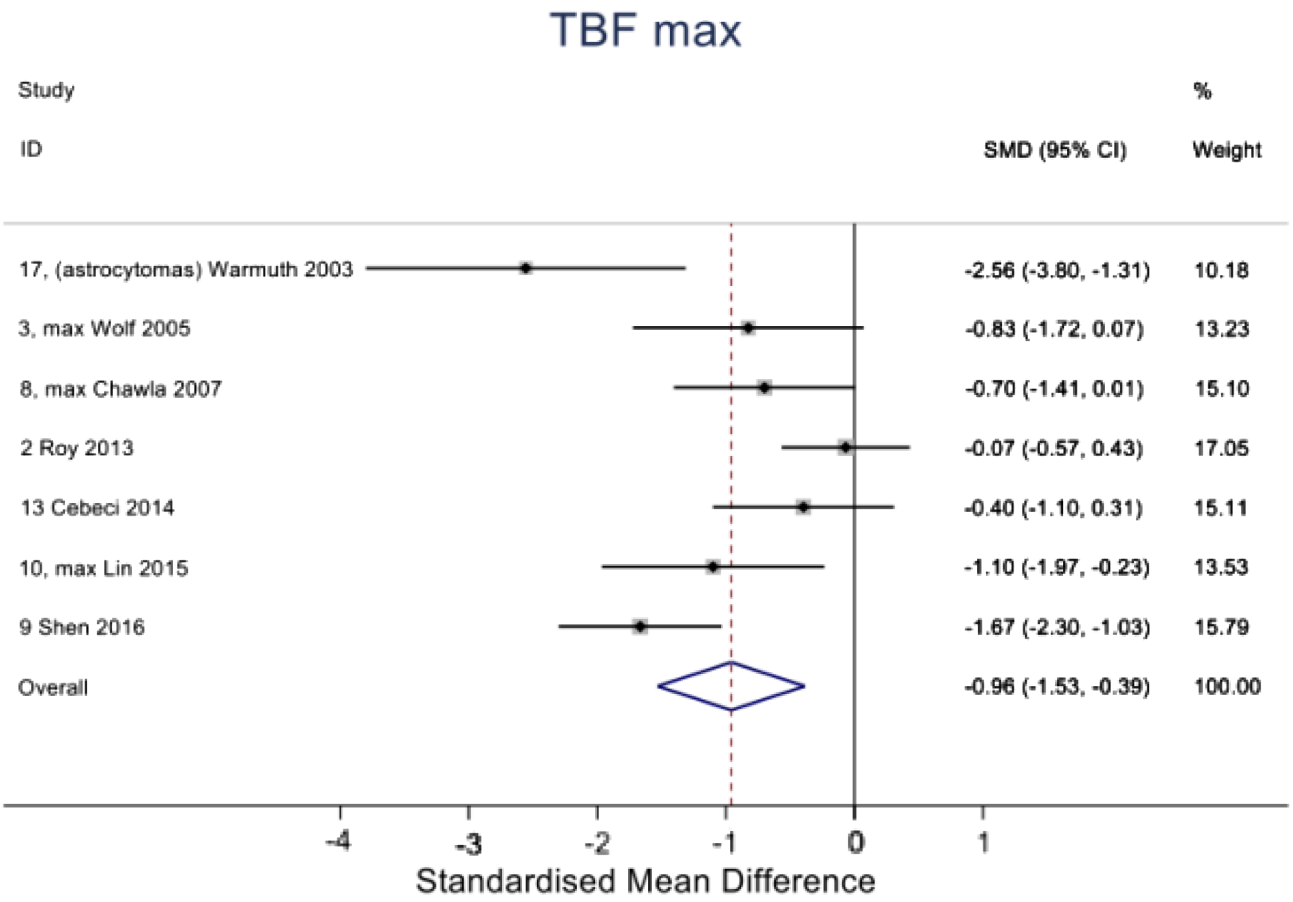

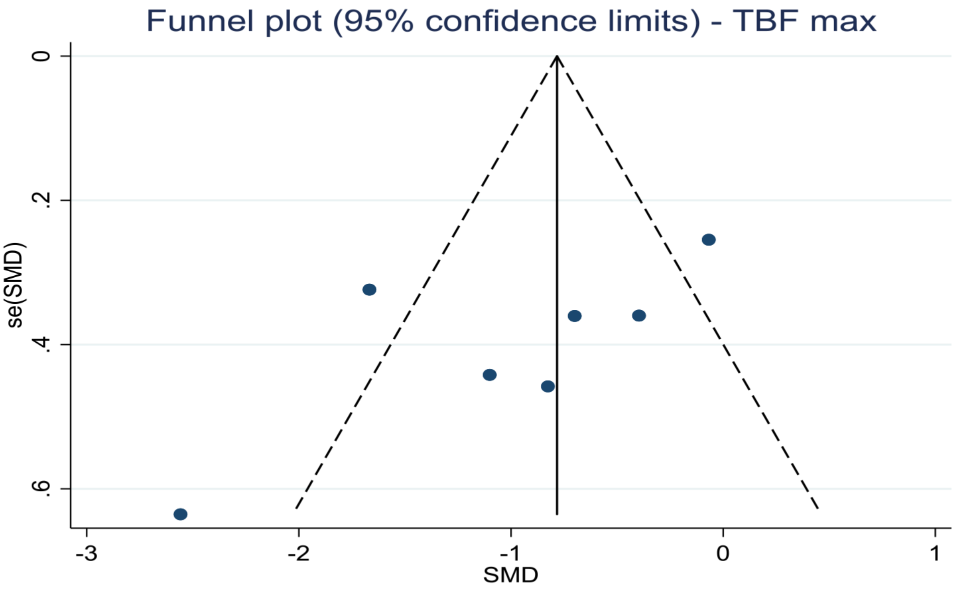

Supplementary Figure 6: TBFmax for LGG patients' relative to the value for HGG patients'. In the forest plot, the dotted vertical line represents the pooled effect size point where the effect size in individual studies have very different distribution (heterogeneity) around this line. The pooled effect and their 95% CI (the diamond at the bottom) express that the LGG have significantly lower TBFmax than the HGG (–0.96, (–1.53, –0.39)). The funnel plot is symmetric and does not show publication bias.

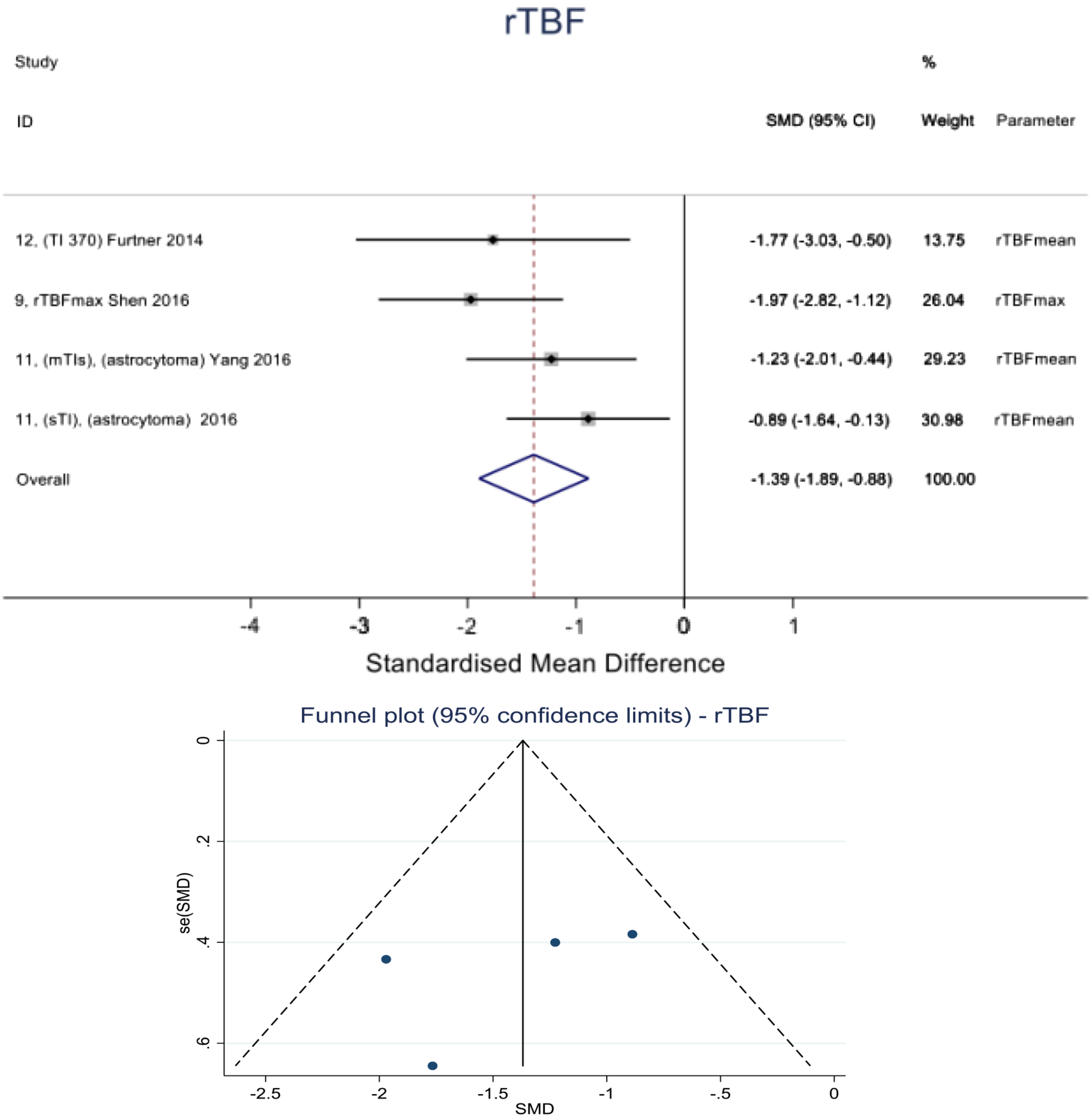

Supplementary Figure 7: rTBF for grade-II patients' relative to the value for grade-III patients'. In the forest plot, the dotted vertical line represents the pooled effect size point where the effect size in individual studies have low distribution (small heterogeneity degree) around this line. The pooled effect and their 95% CI (the diamond at the bottom) express that the grade-II rTBF value about significantly lower than the that of the grade-III (–1.39, (–1.89, –0.89)). The funnel plot is symmetric and does not show publication bias.

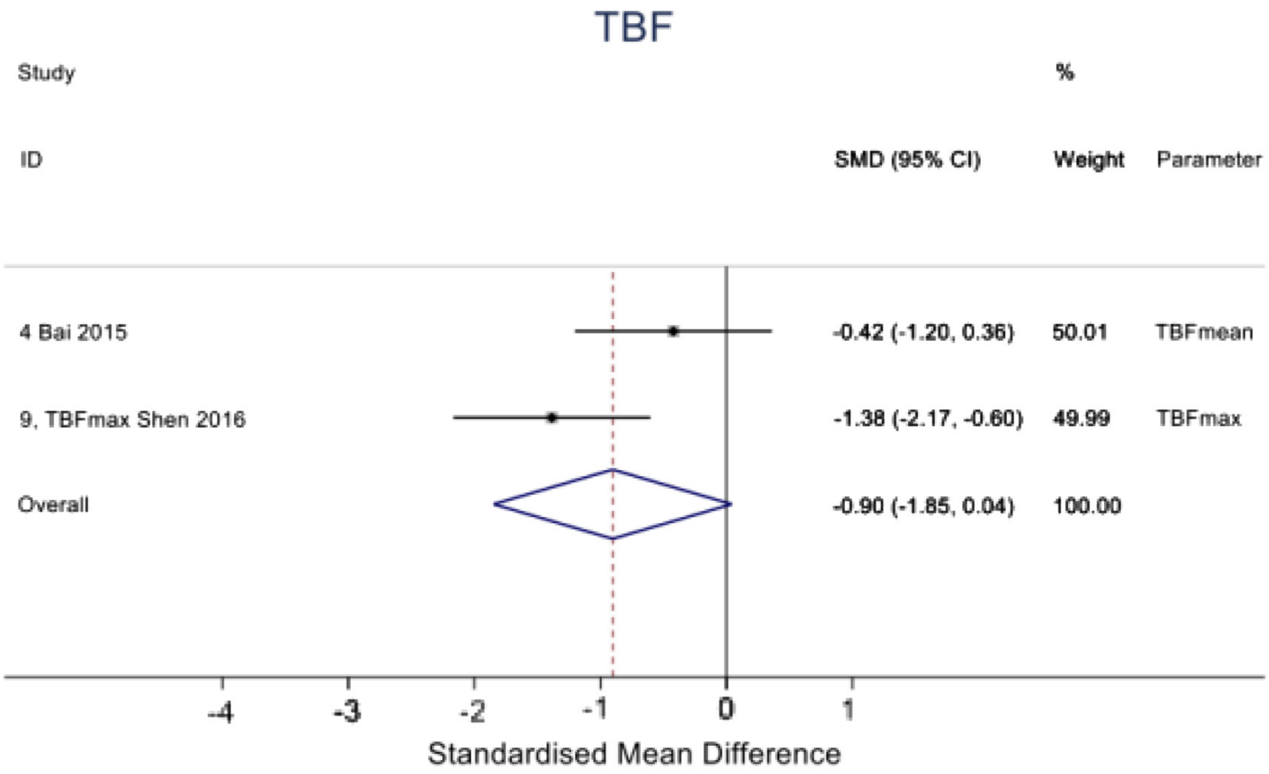

Supplementary Figure 8: TBF for grade-II patients' relative to the value for grade-III patients'. In the forest plot, the dotted vertical line represents the pooled effect size point where the effect size in individual studies have moderate distribution (moderate heterogeneity degree) around this line. The pooled effect and their 95% CI (the diamond at the bottom) express that the grade-II has approximately significant lower TBF value than the grade-III (–0.90, (–1.85, 0.04)). The funnel plot cannot be produced due to the small study number.

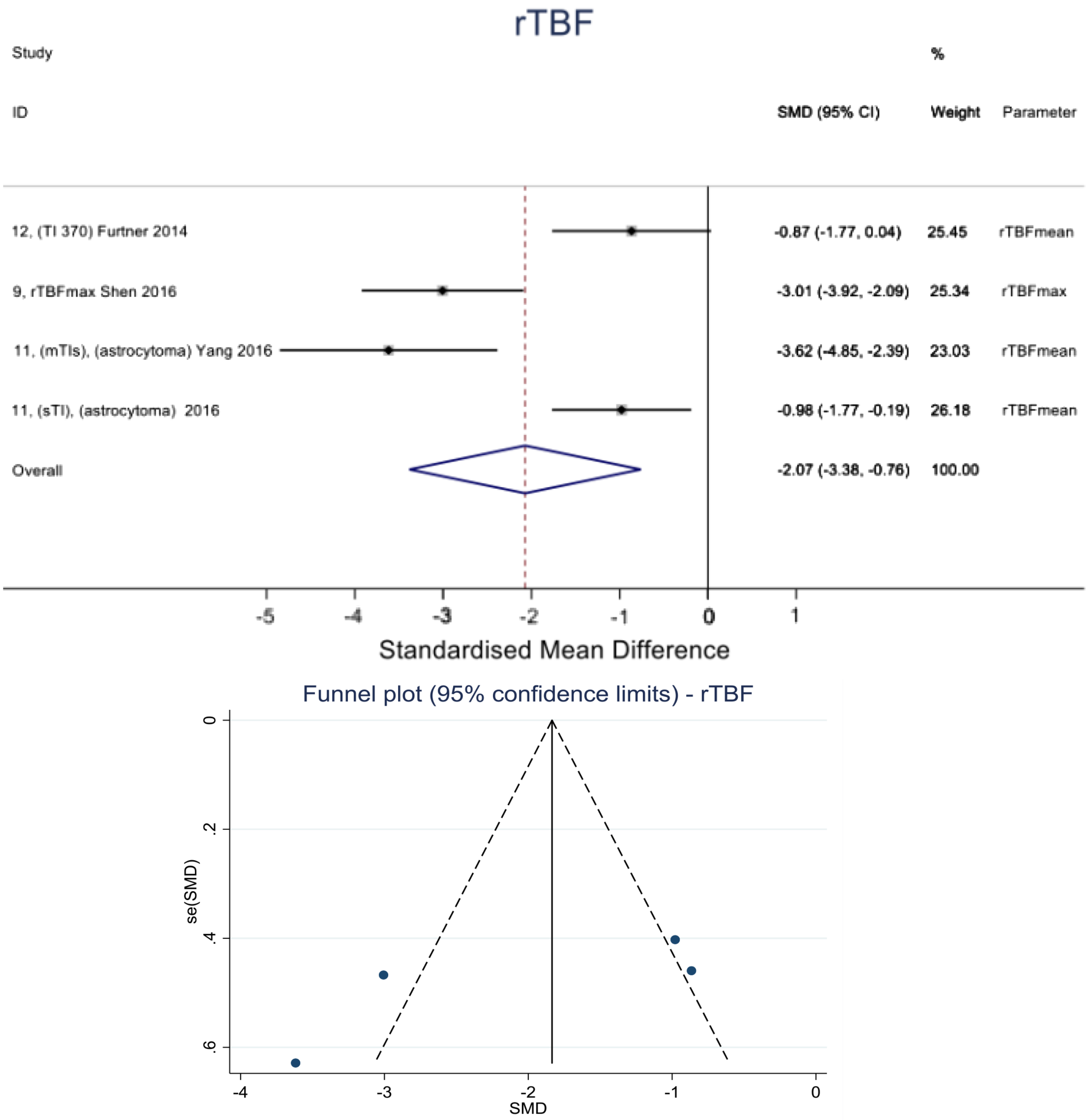

Supplementary Figure 9: rTBF for grade-II patients' relative to the value for grade-IV patients'. In the forest plot, the dotted vertical line represents the pooled effect size point where the effect size in individual studies have very large distribution (heterogeneity) around this line. The pooled effect and their 95% CI (the diamond at the bottom) express that the grade-II rTBF value was significantly lower than the that of the grade-IV (–2.07, (–3.38, –0.76)). The funnel plot is symmetric and does not show publication bias.

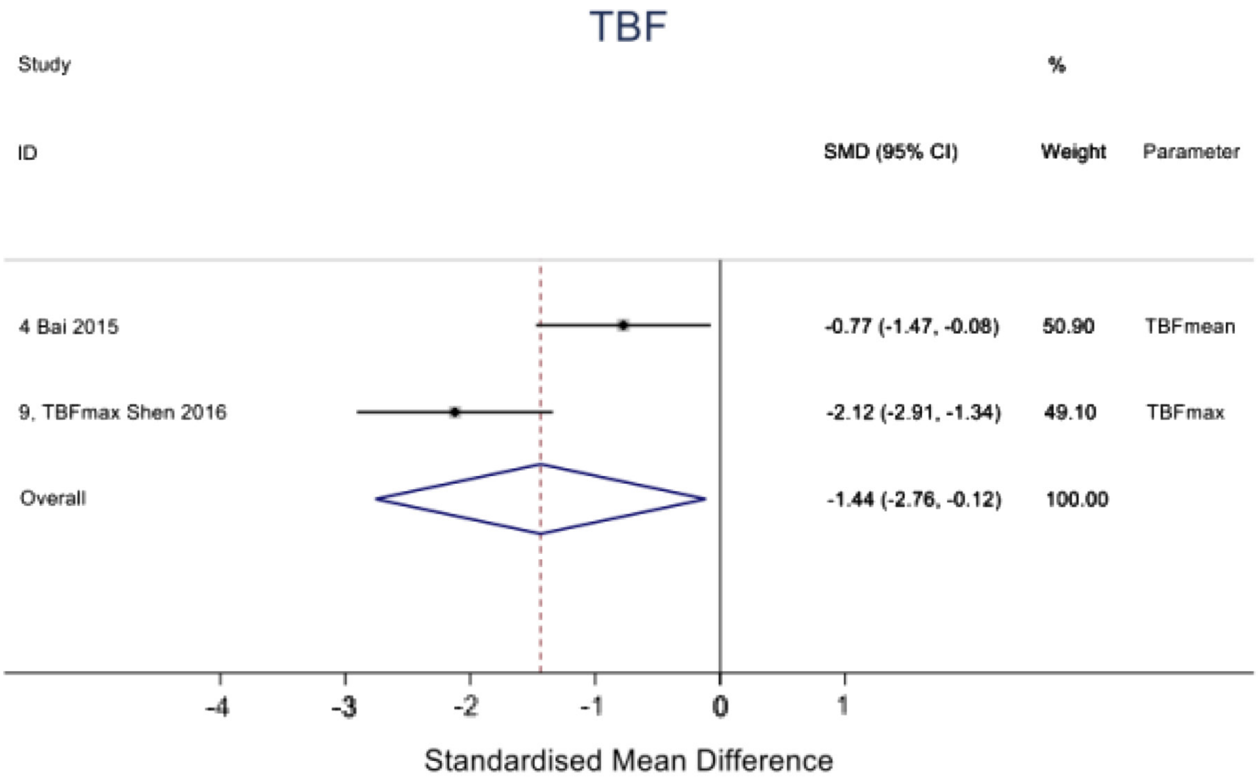

Supplementary Figure 10: TBF for grade-II patients' relative to the value for grade-IV patients'. In the forest plot, the dotted vertical line represents the pooled effect size point where the effect size in individual studies represent very large distribution (heterogeneity) around this line. The pooled effect and their 95% CI (the diamond at the bottom) express that the grade-II significantly lower TBF value than the grade-IV (–1.44, (–2.76, –0.12)). The funnel plot cannot be produced due to the small study number.

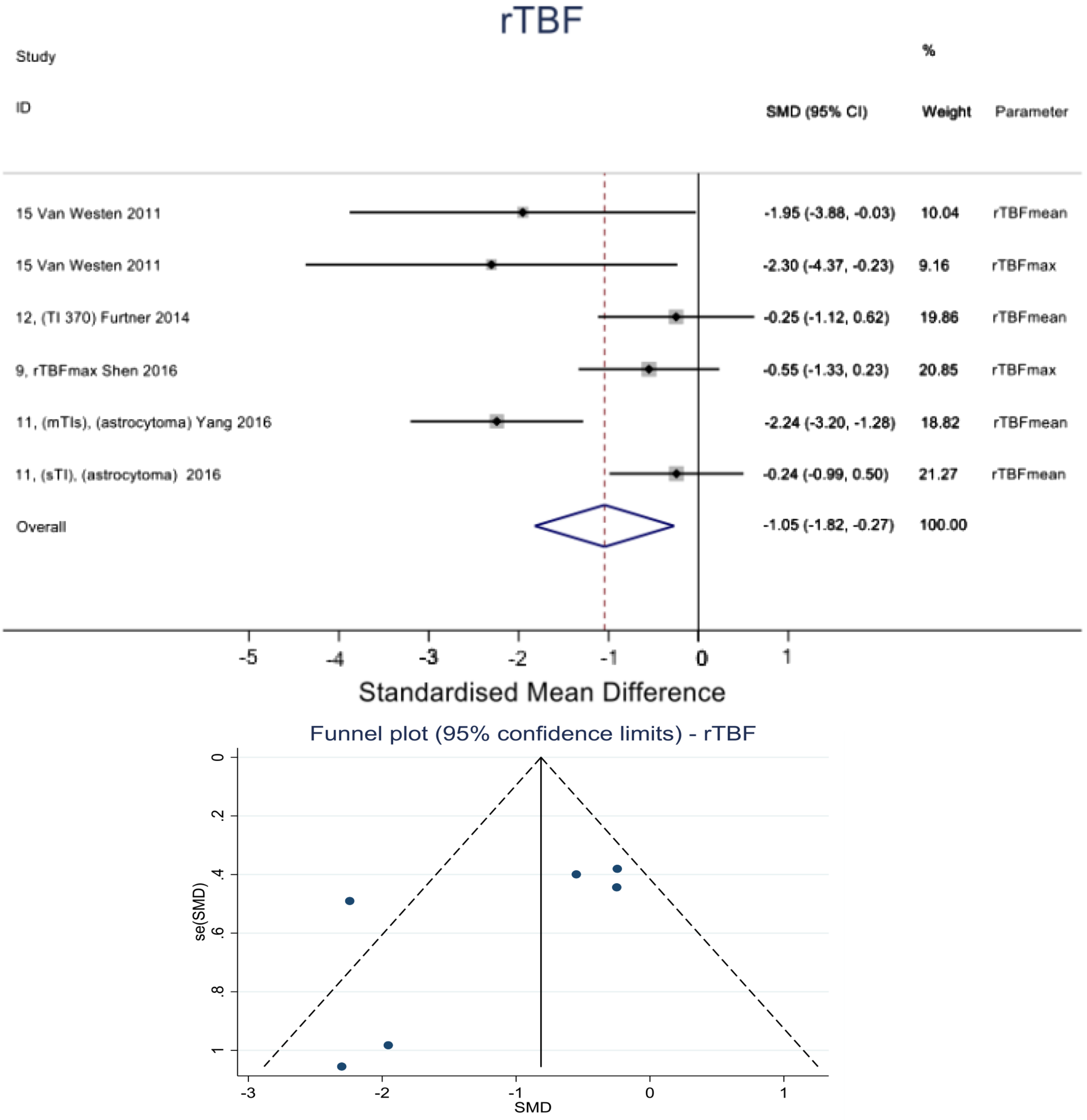

Supplementary Figure 11: rTBF for grade-III patients' relative to the value for grade-IV patients'. In the forest plot, the dotted vertical line represents the pooled effect size point where the effect size in individual studies have large distribution (heterogeneity) around this line. The pooled effect and their 95% CI (the diamond at the bottom) express that the grade-III significantly has lower rTBF value than the grade-IV (–1.05, (–1.82, –0.27)). The funnel plot is symmetric and does not show publication bias.

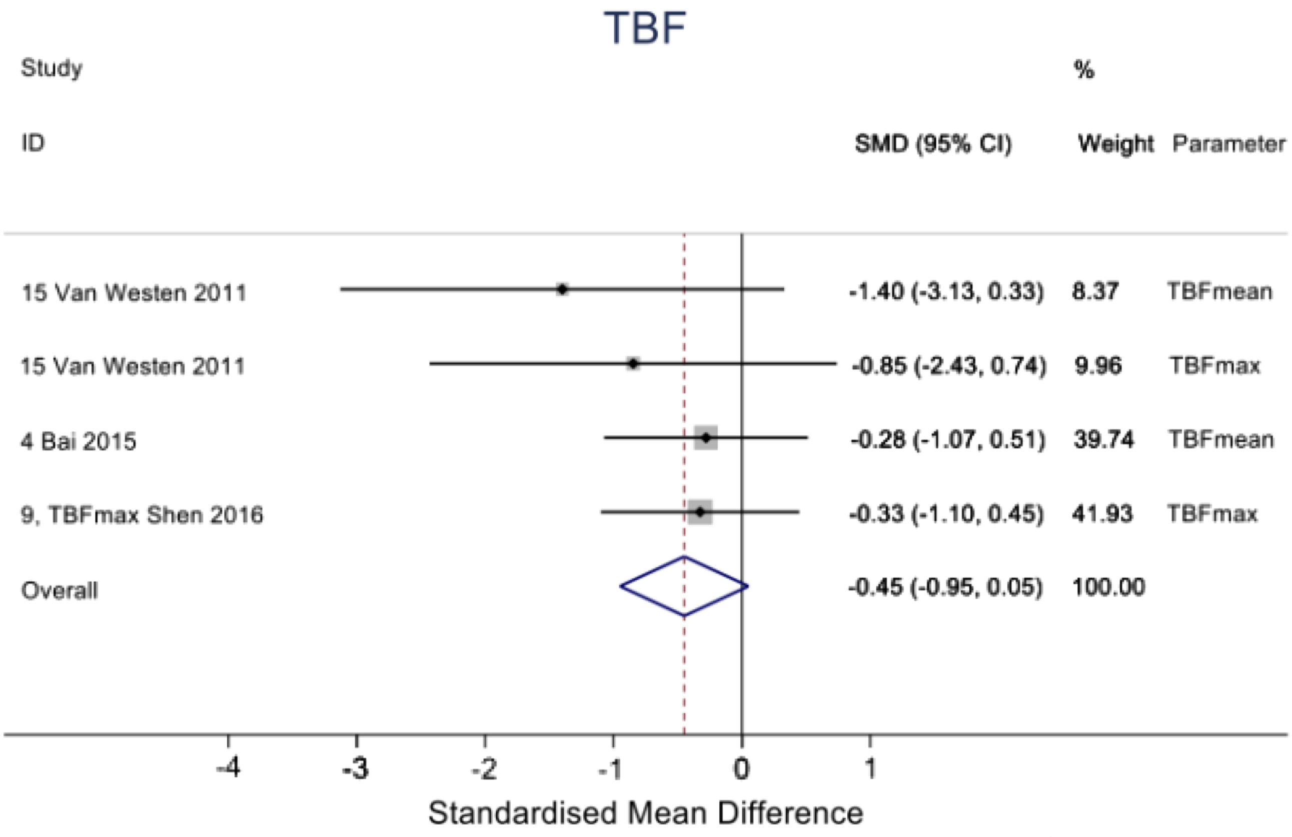

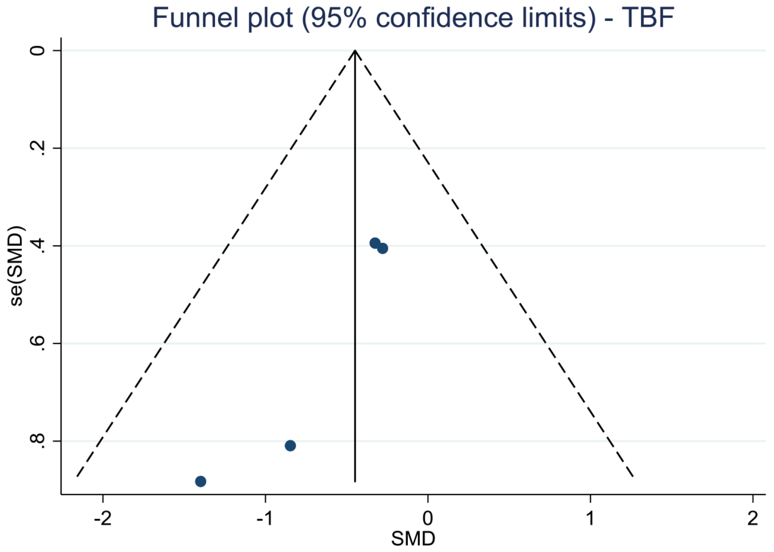

Supplementary Figure 12: TBF for grade-III patients' relative to the value for grade-IV patients'. In the forest plot, the dotted vertical line represents the pooled effect size point where the effect size in individual studies have low distribution (small heterogeneity degree) around this line. The pooled effect and their 95% CI (the diamond at the bottom) express a trend of lower rTBF value in grade-III than in grade-IV (–0.45, (–0.95, 0.05)). The funnel plot is asymmetric and does show publication bias.

Supplementary Table 1: Studies performed using PCASL

Study no.	Authors	Publication year	Country of origin	Gliomas type Oligodendrogliomas/ astrocytomas/mixed	LGGs Grade I	LGGs Grade II	HGGs Grade III	HGGs Grade IV	Histologic analysis obtained with	Study design	MRI field strength	2D/3D	Bolus width (ms)	TI/PLD (ms)	Examined perfusion metrics	Significance for differentiation between HGGs and LGGs
2[1]	Roy, B. et al.	2013	India	indistinct	3	23	9 astrocytoma	29 GBM	NA	prospective	3T	3D	1450	1525	TBFmax<br>rTBFmax	$P = 0.78$<br>$P = 0.12$
4[2]	Bai, Y. et al.	2015	USA	mixed	NA	18(13 astrocytoma, 2 oligodendroglioma)	10(5 anaplastic astrocytoma, 2 anaplastic oligodendroglioma, 3 anaplastic oligo-astrocytoma)	16 GBM	Surgical resection	prospective	3T	3D	2025	1525	TBFmean	Grade-II / III, $p = 0.874$<br>Grade-II / IV, $p = 0.023$<br>Grade-III/ IV, $p = 0.213$
9[3]	Shen, N. et al.	2016	China	mixed	25 (19 astrocytoma, 6 oligodendroglioma)		27 (10 anaplastic astrocytoma, 1 anaplastic oligodendroglioma, 16 GBM)		NA	prospective	3T	3D	1500	1525	TBFmax<br>rTBFmax	$P < 0.001$, including sub-grading ($P < 0.001$)<br>$P < 0.001$, including sub-grading ($P < 0.001$)
10[4]	Lin, Y. et al.	2015	China	mixed	11 (7 diffuse astrocytoma, 3 oligodendroglioma, 1 capillary astrocytoma)		8 anaplastic astrocytoma	5 GBM		prospective	3T	3D	Not mentioned	1500 TBFmax	TBFmean	$P = 0.011$<br>$P = 0.002$
(*)18[5]	Gao, F. et al.	2015	China	indistinct	28		21		NA	prospective	3T	2D	Not mentioned	1400	rTBFmean (WM)<br>rTBFmean (GM)<br>rTBFmean (mirror)	$P < 0.001$<br>$P < 0.001$<br>$P < 0.001$

(*)study 18 did not mentioned the used ASL labelling method.
Not available (NA).

Supplementary Table 2: Studies performed using CASL

Study no.	Author	Publication year	Country of origin	Gliomas type Oligodendrogliomas/ astrocytomas/mixed	LGGs Grade I	LGGs Grade II	HGGs Grade III	HGGs Grade IV	Histologic analysis obtained with	Study design	MRI field strength	2D/3D	Bolus width (ms)	TI/ PLD (ms)	Examined perfusion metrics	Significance for differentiation between HGGs and LGGs
3[6]	Wolf, R. et al.	2005	USA	mixed	2 (ganglioma)	5 (1 oligodendroglioma, 1 astrocytoma, 3 oligoastrocytoma)	8 (1 anaplastic oligodendroglioma, 4 anaplastic astrocytoma, 3 anaplastic oligoastrocytoma)	11 GBM	NA	NA	3T	2D	2000	1200	TBFmean<br>TBFmax<br>rTBFmean<br>rTBFmax	$P = 0.39$<br>$P = 0.04$<br>$P = 0.06$<br>$P = 0.01$
				mixed							3T	3D	2000	1200	TBFmax<br>TBFmean<br>rTBFmean<br>rTBFmax	$P < 0.05$<br>$P > 0.05$<br>$P > 0.05$<br>$P > 0.05$
8[7]	Chawla, S. et al.	2007	USA		1 gabglioma	12 (1 astrocytoma, 11 oligodendroglioma)	9 (4 astrocytoma, 5 oligodendroglioma)	13 GBM	NA	retrospective						
				Oligodendrogliomas											rTBFmax	$P > 0.05$
16[8]	Canale, S. et al.	2011	France	indistinct	NA	5 oligodendroglioma	11 (9 oligodendroglioma, 2 TGNM)	5 GBM	NA	retrospective	1.5T	3D	Not mentioned	1200	rTBFmean	yes

Not available (NA).

Supplementary Table 3: Studies performed using PASL

Study no.	Author	Publication year	Country of origin	Gliomas type Oligodendrogliomas/ astrocytomas/mixed	LGGs		HGGs		Histologic analysis obtained with	Study design	PASL aproach	MRI field strength	2D/3D	Bolus width (ms)	TI/PLD (ms)	Examined perfusion metrics	Significance for differentiation between HGGs and LGGs
					Grade I	Grade II	Grade III	Grade IV									
1[9]	Cornelia B. et al.	2017	Germany	mixed	NA	20(7 oligoden droglioma II, 14 astrocytoma II)	11(2 oligodendro glioma III, 9 astrocytoma III)	5 GBM	NA	retrospective	PICORE	3T	2D	700	1800	TBFmean	$P = 0.1030$
5[10]	Kim, H.S. et al.	2007	Korea	indistinct	NA	11	7	15	Surgery or stereotactic biopsy	prospective	FAIR	1.5T	2D	indistinct	1200	ROC-analysis	indistinct
6[11]	Weber, M. et al.	2006	Germany	indistinct	NA	9	11 anaplastic gliomas	35 GBM	stereotactic biopsy	prospective	Not mentioned	1.5	2D	1000	1200	ROC-analysis	indistinct
7[12]	Fudaba, H. et al.	2014	Japan	Both Mix and astrocytomas	NA	9 (3 diffuse astrocytoma, 3 oligodendro glioma and 3 oligoastro cytoma)	8 (3 anaplastic astrocytoma, 4 anaplastic oligodendro glioma, 1 oligoastro cytioma)	15 (14 GBM, 1 GBM with oligodendro glioma component)	Surgery or stereotactic biopsy	retrospective	Not mentioned	3T	2D	Not mentioned	1800	ROC-analysis	indistinct
11[13]	Yang, X. et al.	2016	China	astrocytomas	NA	15 diffuse astrocytoma	15 anaplastic astrocytoma	13 GBM	NA	prospective	FAIR	3T	3D	700	1920	rTBFmean (sTI)<br><br>rTBFmean (mTIs)	HGG vs LGG, $P = 0.003$<br>II vs III, $P = 0.098$<br>II vs IV, $P = 0.006$<br>III vs IV, $P = 0.0905$<br>HGG vs LGG, $P < 0.001$<br>II vs III, $P = 0.021$<br>II vs IV, $P < 0.001$<br>III vs IV, $P = 0.023$
12[14]	Furtner, J. et al.	2014	Austria	astrocytomas	NA	7 (diffuse astrocytoma)	7 (anplastic astrocytoma)	19 GBM	Surgery or stereotactic biopsy	prospective	PICORE	3T	2D	Not mentioned	370	rTBFmean	$P = 0.003$
13[15]	Cebeci, H. et al.	2014	Turkey	mixed	13 (11 oligodendroglioma, 1 disembryoblastic neuroepithelial tumour (DNET), 1 pilocytic astrocytoma)		20 (18 GBM, 1 astrocytoma, 1 gliosarcoma)		NA	retrospective	EPISTAR	3T	2D	Not mentioned	Not mentioned	TBFmax<br>rTBFmax	$P < 0.001$<br>$P < 0.001$
14[16]	Kim, M J. et al.	2008	Korea	astrocytomas	NA	26	12 anapalstic astrocytomas	23 GBM	Surgery or stereotactic biopsy	prospective	FAIR	1.5T	2D	Not mentioned	1200	rTBFmax	$P < 0.05$
15[17]	Van, W. et al.	2011	Sweden	indistinct	NA	NA	3	4	Biopsy proven	NA	QUASAR	3T	2D	Not mentioned	Not mentioned	TBFmax<br>TBFmean<br>rTBFmax<br>rTBFmean	Just mentioned the trend of increasing the TBF from grade-III towards grade-IV
16[8]	Canale, S. et al.	2016	France	indistinct	NA	3 oligodendro glioma	12 (9 oligodendro glioma III, 3 TGNM)	4 GBM	NA	retrospective	Not mentioned	1.5T	2D	Not mentioned	1200	rTBFmean	Using PASL, $P > 0.05$<br>Using CASL, $P < 0.05$
17[18]	Warmuth, C. et al.	2003	Germany	mixed	3 (2 ganglioma, 1 pleomorphic xanthoastrocytoma)	6 (5 astro cytoma, 1 optic astrocytoma)	3 (1 anaplastic oligodendro glioma, 1 anaplastic astrocytoma, 1 astrocytoma)	7 GBM	NA	prospective	FAIR	1.5T	2D	1200	1300	TBFmax<br>rTBFmax	$P < 0.001$<br>$P < 0.001$

Not available (NA); Proximal Inversion with Control of Off-Resonance Effects (PICORE); flow alternating inversion recovery (FAIR); quantitative STAR labeling of arterial regions (QUASAR).

Supplementary Table 4: Sensitivity, specificity, negative predictive values (NPV) and positive predictive values (PPV) of published ASL-derived biomarkers cut-off values for glioma grading

II vs III gliomas

Author/year	Study No.	ASL parameters	Cut-off	Sensitivity	Specificity	Prevalence of grade III	PPV	NPV
Weber, M A. et al.; 2006	6	rTBFmean	1	0.92	0.33	0.55	62.66	77.14
Shen, N. et al.; 2016	9	TBFmax	43.62	1	0.69	0.31	59.12	100
Yang, X. et al.; 2016	11, (multiple TIs), (astrocytoma)	rTBFmean	2.43	1	0.51	0.5	67.11	100
	11, (single TI), (astrocytoma)	rTBFmean	1.88	0.78	0.73	0.5	74.29	76.84

II vs IV gliomas

Author/year	Study No.	ASL parameters	Cut-off	Sensitivity	Specificity	Prevalence of grade-IV	PPV	NPV
Weber, M A. et al.; 2006	6	rTBFmean	1.6	0.94	0.78	0.80	94.32	76.97
Yang, X. et al.; 2016	11, (multiple TIs), (astrocytoma)	rTBFmean	4	1	0.87	0.46	86.96	100
	11, (single TI), (astrocytoma)	rTBFmean	3.01	0.67	0.87	0.46	81.71	75.26

III vs IV gliomas

Author/year	Study No.	ASL parameters	Cut-off	Sensitivity	Specificity	Prevalence of grade-IV	PPV	NPV
Weber, M A. et al.; 2006	6	rTBFmean	1.4	0.97	0.5	0.76	86.06	83.97
Fudaba, H. et al.; 2014	7	rTBFmean	2.562	0.87	0.77	0.65	87.37	75.42
		rTBFmax	2.845	0.87	0.82	0.65	90.23	76.77
		rTBFmin	2.017	0.87	0.59	0.652173913	79.78038157	70.21943574
	7, (astrocytoma)	rTBFmean	1.857	0.93	0.83	0.82	96.29	71.54
	7, (astrocytoma)	rTBFmax	2.258	0.93	0.83	0.82	96.29	71.54
	7, (astrocytoma)	rTBFmin	2.164	0.79	0.83	0.824	95.645	45.478
Yang, X. et al.; 2016	11, (multiple TIs), (astrocytoma)	rTBFmean	8.55	0.77	0.73	0.464	71.19	78.55
	11, (single TI), (astrocytoma)	rTBFmean	6.64	0.46	0.73	0.464	59.62	60.93

Supplementary Table 5: Sensitivity, specificity, negative predictive values (NPV) and positive predictive values (PPV) of published ASL-derived biomarkers cut-off values between HGGs and LGGs

HGG vs LGG								
**Author/ year**	**Study No.**	**ASL parameter**	**Cut-off**	**Sensitivity**	**Specificity**	**prevalence**	**PPV**	**NPV**
Kim, H.S. et al.; 2007	5	rTBFmean	1.24	0.955	0.818	0.667	91.30	90.088
Fudaba, H. et al.; 2014	7	rTBFmean	2.562	0.652	0.778	0.719	88.243	46.661
	7	rTBFmax	2.845	0.609	0.778	0.719	87.516	43.776
	7	rTBFmin	2.017	0.739	0.667	0.719	85.0105	50
	7, (astrocytoma)	rTBFmean	1.8	0.824	0.667	0.85	93.343	40.076
	7, (astrocytoma)	rTBFmax	2.258	0.765	0.667	0.85	92.866	33.372
	7, (astrocytoma)	rTBFmin	1.254	0.882	0.667	0.85	93.753	49.937
Shen, N. et al.; 2016	9	TBFmax	52.21	0.889	0.826	0.5192	84.664	87.317
	9	rTBFmax	1.32	0.926	0.957	0.519	95.831	92.279
Yang, X. et al.; 2016	11, (multiple TIs), (astrocytoma)	rTBFmean	2.43	1	0.54	0.6511	80.229	100
	11, (single TI), (astrocytoma)	rTBFmean	3.01	0.6	0.88	0.651	90.323	54.098
	11, (bolus arrival time (BAT)), (astrocytoma)		0.97	0.71	0.88	0.651	91.697	61.914
Furtner, J. et al.; 2014	12		1.48	0.85	1	0.788	100	64.220
Cebeci, H. et al.; 2014	13	rTBFmax	2.1	1	0.92	0.606	95.057	100
	13	rSImax	2.19	1	0.92	0.606	95.057	100
Kim, M J. et al.; 2008	14, (astrocytoma)	rTBFmax	1.28	0.829	0.962	0.5738	96.707	80.691
Canale, S. et al.; 2011	16, (oligodendroglioma)	rTBFmean	1.8	0.88	0.6	0.762	87.562	60.976

REFERENCES

1. Roy B, Awasthi R, Bindal A, Sahoo P, Kumar R, Behari S, Ojha BK, Husain N, Pandey CM, Rathore RK, Gupta RK. Comparative evaluation of 3-dimensional pseudocontinuous arterial spin labeling with dynamic contrast-enhanced perfusion magnetic resonance imaging in grading of human glioma. J Comput Assist Tomogr. 2013; 37:321–6. https://doi.org/10.1097/RCT.0b013e318282d7e2.
2. Bai Y, Lin Y, Zhang W, Kong L, Wang L, Zuo P, Vallines I, Schmitt B, Tian J, Song X, Zhou J, Wang M. Noninvasive amide proton transfer magnetic resonance imaging in evaluating the grading and cellularity of gliomas. Oncotarget. 2017; 8:5834–5842. https://doi.org/10.18632/oncotarget.13970.
3. Shen N, Zhao L, Jiang J, Jiang R, Su C, Zhang S, Tang X, Zhu W. Intravoxel incoherent motion diffusion-weighted imaging analysis of diffusion and microperfusion in grading gliomas and comparison with arterial spin labeling for evaluation of tumor perfusion. Journal of Magnetic Resonance Imaging. 2016. 620–32. https://doi.org/10.1002/jmri.25191.
4. Lin Y, Li J, Zhang Z, Xu Q, Zhou Z, Zhang Z, Zhang Y, Zhang Z. Comparison of intravoxel incoherent motion diffusion-weighted MR imaging and arterial spin labeling MR imaging in gliomas. Biomed Res Int. 2015; 2015:234245. https://doi.org/10.1155/2015/234245.
5. Gao F, Guo R, Hu XJ, Li CJ, Li M. Noninvasive Tumor Grading of Glioblastomas Before Surgery Using Arterial Spin Labeling. A Cohort Study. Anal Quant Cytopathol Histopathol. 2015; 37:339–46.
6. Wolf RL, Wang J, Wang S, Melhem ER, O’Rourke DM, Judy KD, Detre JA. Grading of CNS neoplasms using continuous arterial spin labeled perfusion MR imaging at 3 Tesla. J Magn Reson Imaging. 2005; 22:475–82. https://doi.org/10.1002/jmri.20415.
7. Chawla S, Wang S, Wolf RL, Woo JH, Wang J, O’Rourke DM, Judy KD, Grady MS, Melhem ER, Poptani H. Arterial spin-labeling and MR spectroscopy in the differentiation of gliomas. Am J Neuroradiol. 2007; 28:1683–9. https://doi.org/10.3174/ajnr.A0673.
8. Canale S, Rodrigo S, Tourdias T, Mellerio C, Perrin M, Souillard R, Oppenheim C, Meder JF. [Grading of adults primitive glial neoplasms using arterial spin-labeled perfusion MR imaging]. [Article in French]. J Neuroradiol. 2011; 38:207–13. https://doi.org/10.1016/j.neurad.2010.12.003.
9. Brendle C, Hempel JM, Schittenhelm J, Skardelly M, Tabatabai G, Bender B, Ernemann U, Klose U. Glioma Grading and Determination of IDH Mutation Status and ATRX loss by DCE and ASL Perfusion. Clinical Neuroradiology. 2017. https://doi.org/10.1007/s00062-017-0590-z.
10. Kim HS, Kim SY. A prospective study on the added value of pulsed arterial spin-labeling and apparent diffusion coefficients in the grading of gliomas. Am J Neuroradiol. 2007; 28:1693–9. https://doi.org/10.3174/ajnr.A0674.
11. Weber MA, Zoubaa S, Schlieter M, Juttler E, Huttner HB, Geletneky K, Ittrich C, Lichy MP, Kroll A, Debus J, Giesel FL, Hartmann M, Essig M. Diagnostic performance of spectroscopic and perfusion MRI for distinction of brain tumors. Neurology. 2006; 66:1899–906. https://doi.org/10.1212/01.wnl.0000219767.49705.9c.
12. Fudaba H, Shimomura T, Abe T, Matsuta H, Momii Y, Sugita K, Ooba H, Kamida T, Hikawa T, Fujiki M. Comparison of multiple parameters obtained on 3T pulsed arterial spin-labeling, diffusion tensor imaging, and MRS and the Ki-67 labeling index in evaluating glioma grading. AJNR Am J Neuroradiol. 2014; 35:2091–8. https://doi.org/10.3174/ajnr.A4018.
13. Yang XS, Zhao B, Wang G, Xiang J, Xu S, Liu Y, Zhao P, Pfeuffer J, Qian T. Improving the grading accuracy of astrocytic neoplasms noninvasively by combining timing information with cerebral blood flow: A multi-Ti arterial spin-labeling MR imaging study. AJNR Am J Neuroradiol. 2016; 37:2209–16. https://doi.org/10.3174/ajnr.A4907.
14. Furtner J, Schöpf V, Schewzow K, Kasprian G, Weber M, Woitek R, Asenbaum U, Preusser M, Marosi C, Hainfellner JA, Widhalm G, Wolfsberger S, Prayer D. Arterial spin-labeling assessment of normalized vascular intratumoral signal intensity as a predictor of histologic grade of astrocytic neoplasms. AJNR Am J Neuroradiol. 2014; 35:482–9. https://doi.org/10.3174/ajnr.A3705.
15. Cebeci H, Aydin O, Ozturk-Isik E, Gumus C, Inecikli F, Bekar A, Kocaeli H, Hakyemez B. Assesment of perfusion in glial tumors with arterial spin labeling; comparison with dynamic susceptibility contrast method. Eur J Radiol. 2014; 83:1914–9. https://doi.org/10.1016/j.ejrad.2014.07.002.
16. Kim MJ, Kim HS, Kim JH, Cho KG, Kim SY. Diagnostic accuracy and interobserver variability of pulsed arterial spin labeling for glioma grading. Acta Radiol. 2008; 49:450–7. https://doi.org/10.1080/02841850701881820.
17. van Westen D, Petersen ET, Wirestam R, Siemund R, Bloch KM, Ståhlberg F, Björkman-Burtscher IM, Knutsson L. Correlation between arterial blood volume obtained by arterial spin labelling and cerebral blood volume in intracranial tumours. MAGMA. 2011; 24:211–23. https://doi.org/10.1007/s10334-011-0255-x.
18. Warmuth C, Günther M, Zimmer C. Quantification of Blood Flow in Brain Tumors: Comparison of Arterial Spin Labeling and Dynamic Susceptibility-weighted Contrast-enhanced MR Imaging. Radiology. 2003; 228:523–32. https://doi.org/10.1148/radiol.2282020409.
